# Rebound Bursting Selectively Enables Fast Dynamics in Dopamine Midbrain Neurons Projecting to the Dorsolateral Striatum

**DOI:** 10.1523/JNEUROSCI.0361-25.2025

**Published:** 2025-09-26

**Authors:** Strahinja Stojanovic, Christopher J. Knowlton, Richard Egger-Mackrodt, Johanna Mankel, Josef Shin, Stephan Lammel, Carmen C. Canavier, Jochen Roeper

**Affiliations:** ^1^Institute of Neurophysiology, Neuroscience Center, Goethe University, Frankfurt am Main 60590, Germany; ^2^Department of Cell Biology and Anatomy, School of Medicine, Louisiana State University Health Sciences Center, New Orleans, Louisiana 70112; ^3^Department of Neuroscience, Helen Wills Neuroscience Institute, University of California, Berkeley, California 94720

**Keywords:** burst, Cav3 channel, DA, SK channel, substantia nigra, T-type channel

## Abstract

Dopamine (DA) midbrain neurons are involved in a wide array of key brain functions including movement control and reward-based learning. They are also critical for major brain disorders such as Parkinson’s disease or schizophrenia. DA neurons projecting to distinct striatal territories are diverse with regard to their molecular makeup and cellular physiology, which are likely to contribute to the observed differences in temporal DA dynamics. Among these regions, the dorsolateral striatum (DLS) displays the fastest DA dynamics, which might control the moment-to-moment vigor and variability of voluntary movements. However, the underlying mechanisms for these DLS-specific fast DA fluctuations are unresolved. Here, we show that DLS-projecting DA neurons in the substantia nigra (SN) possess a unique biophysical profile allowing immediate 10-fold accelerations in discharge frequency via rebound bursting. By using a combination of in vitro patch-clamp recordings in projection-defined DA SN subpopulations from adult male mice and developing matching projection-specific computational models, we demonstrate that a strong interaction of Ca_v_3 and SK channels specific for DLS-projecting Aldh1a1–positive DA SN (DLS-DA) neurons controls the gain of fast rebound bursting, while K_v_4 and HCN channels mediate timing of rebound excitability. In addition, GIRK channels activated by D2 and GABA_B_ receptors prevent rebound bursting in these DLS-DA neurons. Furthermore, our in vivo patch-clamp recordings and matching in vivo computational models provide evidence that these unique rebound properties might be preserved in the intact brain, where they might endow specific computational properties well suited for the generation of fast DA dynamics present in DLS.

## Significance Statement

Dorsolateral striatum (DLS)-projecting dopamine (DA) neurons in the substantia nigra exhibit unique rebound bursting that enables rapid, 10-fold increases in firing frequency. This firing fingerprint is driven by Ca_v_3 and SK channel interactions, modulating burst gain, and fine-tuned by K_v_4 and HCN channels controlling rebound timing. GIRK channels, activated by D2 and GABA_B_ receptors, inhibit this bursting. In vivo patch-clamp recordings provide evidence that these rebound dynamics might be preserved in the intact brain, potentially supporting the fast dopamine fluctuations crucial for controlling movement vigor and variability in the DLS. These findings provide insights into the mechanisms underlying fast DA dynamics and their role in motor function, with implications for brain disorders like Parkinson’s disease and schizophrenia.

## Introduction

Dopamine (DA) midbrain neurons are crucial for essential brain functions ranging from voluntary movement to reward-based learning and cognition (Klaus et al., 2019; de Jong et al., 2022). This is also reflected in major diseases that involve the DA system such as schizophrenia, Parkinson’s disease, and substance abuse disorders (Surmeier et al., 2017; McCutcheon et al., 2019; Wise and Robble, 2020). The diversity of DA functions is realized on multiple scales ranging from gene expression profiles of individual DA neurons (Poulin et al., 2014, 2018; Saunders et al., 2018; Tiklová et al., 2019), cellular electrophysiology, and calcium dynamics (Lammel et al., 2008; Evans et al., 2017; Tarfa et al., 2017) up to differential synaptic connectivity resulting in different network affiliations (Lammel et al., 2012; Watabe-Uchida et al., 2012; Beier et al., 2015, 2019; Lerner et al., 2015; Menegas et al., 2015; Tian et al., 2016).

Recent elegant studies have highlighted important regional striatal differences in DA signaling with exceptionally fast DA dynamics identified in the dorsolateral striatum (DLS). This is likely to control the fast sequencing, vigor, and variability of moment-to-moment motor behavior (Howe and Dombeck, 2016; Azcorra et al., 2023; Jørgensen et al., 2023; Markowitz et al., 2023; Mohebi et al., 2024). In contrast, DA dynamics are much slower in dorsomedial and ventral striatal regions, potentially reflecting slower reward-related processes such as updating of reward prediction errors across different timescales. However, the underlying mechanisms that enable distinct DA subpopulations with variable temporal signaling properties are yet unresolved. Plausible candidate mechanisms range from differences in axonal DA release and uptake (for review, see Sulzer et al., 2016), control of distal axon excitability (Kramer et al., 2020, 2022; Liu et al., 2022), and electrical pattern generation in the somatodendritic domain of DA neurons (Otomo et al., 2020). The latter results from the interaction of synaptic inputs and intrinsic excitability.

The biophysical differences among DA midbrain neurons, which control intrinsic excitability, have been well established, in particular for those in the ventral tegmental area projecting to distinct mesolimbic and prefrontal areas (Lammel et al., 2008, 2011, 2012; de Jong et al., 2019, 2022, 2024). In contrast, DA neurons in the substantia nigra (SN) have until recently often been assumed to be more homogeneous. Lerner and colleagues found only minor intrinsic differences in electrophysiological profiles (e.g., HCN current amplitudes) between DA SN neurons projecting to dorsomedial or dorsolateral striatum (DMS and DLS, respectively; Lerner et al., 2015). However, Evans and colleagues observed strong differences among DA SN neurons regarding their postinhibitory rebound properties (Evans et al., 2017). They showed that calbindin-immunonegative and Aldh1a1-immunopositive DA SN neurons generated stronger low-threshold calcium transients sensitive to Ca_v_3 channel inhibition compared with calbindin-immunopositive DA SN neurons [Evans et al. (2017), their Fig. 3C–E]. In the same study, some calbindin-negative but not Aldh1a1-positive DA SN neurons also displayed rebound bursts (Fig. 3*F*). In addition, we identified the selective role of Ca_v_1.3 channels for DLS-projecting DA SN neurons in amplifying the firing rate across the entire dynamic range (Shin et al., 2022). However, these data do not fully explain how DLS-DA neurons might drive fast DA dynamics.

To address this topic, we combined retrograde tracing, molecular marker expression analysis, in vitro and in vivo patch-clamp recordings, and projection-specific computational modeling to define the electrophysiological differences between DA SN subtypes projecting to DLS, DMS, and the ventral striatum. We identified a unique and highly regulated rebound excitability in DLS-DA neurons as a candidate mechanism for fast DA dynamics in the sensorimotor striatum.

## Materials and Methods

### Animal use

Adult male C57BL/6N (Charles River Laboratories; strain code, 027; https://www.criver.com/products-services/find-model/c57bl6-mouse?region=23) mice (8–12 weeks of age) were used in this study. A maximum of five mice were housed per cage, and single animal housing was avoided when possible. Animals were maintained on a 12 h light/dark cycle and provided with water and food *ad libitum*. All experimental procedures involving mice were approved by the German Regional Council of Darmstadt (V54-19c20/15-FU/1257).

### Stereotactic surgeries

Mice were anesthetized with isoflurane (induction at 4%, maintenance at 1.5–2% in O_2_, 0.35 L/min; Piramal Critical Care) and placed in a stereotaxic frame (Model 1900, David Kopf Instruments). Isoflurane concentration and oxygen flow rate were controlled using inhalation anesthetic vaporizer (Sigma Delta, Penlon). Throughout the procedure, body temperature (37–38°C) and respiratory rate (1–2 Hz) were monitored and kept in the respective target ranges via a custom-made heating blanket and adapting the isoflurane concentration. Eye gel was used for preventing corneal dehydration (Vidisic, Bausch + Lomb). Eutectic mixture of lidocaine and prilocaine (Emla, Aspen) was applied at the scalp as a local analgesic >5 min before the incision was made. Subsequently, retrograde tracing injections or head-plate implantation for in vivo patch-clamp experiments were performed. After the procedure, animals were kept on a heating pad until full recovery from anesthesia. For retrograde tracing, craniotomies were made using a stereotaxic drill (0.6 mm drill tip diameter) to target the DLS (AP, +0.74 mm; ML, 2.2 mm; DV, 2.6 mm), dorsomedial striatum (DMS; AP, +0.74 mm; ML, 1.2 mm; DV, 2.6 mm), and lateral shell of the nucleus accumbens (lNAcc; AP, +0.86 mm; ML, 1.75 mm; DV, 4.5 mm). Correction of the coordinates was done as previously described (Lammel et al., 2008). Red beads (RB; 200 nl; Lumafluor) were diluted (1:30) using artificial cerebrospinal fluid (ACSF; Harvard Apparatus) and infused into the area of interest. The tracer was infused bilaterally into the target area using a syringe (10 µl nanofil syringe, 33 G steel beveled needle; World Precision Instruments) attached to a microinjection pump (flow rate 100 nl/min; UMP3-1 and MICRO2T, World Precision Instruments). After a survival period for sufficient retrograde labeling (DLS and lNAcc 3 d, DMS 4 d), animals were killed for histological analysis of molecular markers or used for in vitro patch-clamp recordings. Additionally, serial analysis of the injection sites was carried out as previously described (Lammel et al., 2008). For head-plate implantation: small indentations were made above the SN (AP, −3.08 mm; ML, 0.9–1.4 mm) as a reference for later craniotomies. Custom-made head-plates were mounted on the skull with screws and further fastened using a superglue and dental cement (Paladur, Kulzer).

### Slice preparation and in vitro patch-clamp recordings

On a recording day, mice were terminally anesthetized by ketamine (250 mg/kg; Ketaset, Zoetis) and medetomidine hydrochloride (2.5 mg/kg; Domitor, Orion Pharma) mixture administered intraperitoneally. Once an areflexic state was achieved, the animal was perfused intracardially with ice-cold ACSF containing the following (in mM): 50 sucrose, 125 NaCl, 2.5 KCl, 25 NaHCO_3_, 1.25 NaH_2_PO_4_, 2.5 glucose, 6 MgCl_2_, 0.1 CaCl_2_, and 2.96 kynurenic acid (Sigma-Aldrich). The 250-µm-thick coronal slices of the rostral midbrain (AP, −2.92 to −3.16 mm) were sectioned using a vibrating blade microtome (speed, 0.06 mm/s; amplitude, 1 mm; VT1200s, Leica Biosystems). To allow recovery, slices were kept for 1 h in an extracellular solution consisting of (in mM) 22.5 sucrose, 125 NaCl, 3.5 KCl, 25 NaHCO_3_, 1.25 NaH_2_PO_4_, 2.5 glucose, 1.2 MgCl_2_, and 1.2 CaCl_2_, continuously oxygenated with 95% O_2_/5% CO_2_ at 37°C. After recovery, slices were transferred into a heated (37°C) recording chamber (Luigs & Neumann) and perfused continuously at the flow rate of 2–4 ml/min (resulting in a bath ACSF temperature above the slice of 31–33°C). To inhibit fast excitatory and inhibitory synaptic transmission, 20 µm CNQX (6-cyano-7-nitroquinoxaline-2,3-dione), 4 µm gabazine (SR95531), and 10 µm DL-AP5 were added. A combination of 1 µm oxotremorine-M, 10 µm phenylephrine, 600 nm sulpiride, and 50 nm CGP 55845 was used to desensitize muscarinergic and noradrenergic and inhibit D2 AR- and GABA_B_-mediated signaling, respectively (when drugs were used separately, it was indicated in the Results section). Neurons were visualized using an infrared differential interference contrast videomicroscopy with a digital camera (VX55, Till Photonics) mounted on an upright microscope (Axioskop 2, Carl Zeiss). Retrogradely labeled neurons were identified by excitation of RB using an epifluorescence illumination light source at 546/12 nm (X-cite 120PC Q, Excelitas Technologies). Patch pipettes (4–6 MΩ) were pulled from borosilicate glass (GC150TF-10, 1.50 OD × 1.17 ID × 100 L mm, Harvard Apparatus) using a horizontal pipette puller (DMZ-Universal-Electrode-Puller, Zeitz-Instruments). Patch pipettes were filled with internal solution containing the following (in mM): 135 K-gluconate, 5 KCl, 10 HEPES, 0.1 EGTA, 5 MgCl_2_, 0.075 CaCl_2_, 5 NaATP, 1 LiGTP, and 0.1% neurobiotin (NB), pH 7.35–7.4, (adjusted using KOH; osmolarity, 290–300 mOsm). To alter intracellular calcium buffering, three additional internal solutions were used. One solution was identical in composition except that K-gluconate was replaced with K-methanesulfonate (135 mM KMeSO₃) while maintaining 0.1 mM EGTA and 0.075 mM CaCl₂. The other two solutions were based on the K-gluconate internal solution but contained either 1 mM EGTA or 1 mM BAPTA, with CaCl₂ omitted to minimize free intracellular calcium. Estimated free Ca^2+^ concentrations for each internal solution were calculated using the MaxChelator program and are summarized in Table 4. The data presented were not corrected for the liquid junction potential (ca. 10 mV for K-gluconate solutions). Besides the liquid junction potential, an additional voltage error arises from measuring the membrane potential across the combination of membrane resistance (Rm) and series resistance (Rs) in series (voltage divider). The fraction of voltage dropping across Rs depends on the ratio Rs/(Rs + Rm) (Horowitz et al., 1989; Axon Instruments Inc., 1993). Using typical values for in vitro (Rs ≈ 10 MΩ; Rm ≈ 1,000 MΩ) and in vivo (Rs ≈ 30 MΩ; Rm ≈ 300 MΩ) conditions, this results in an error of ∼1% in vitro and ∼10% in vivo. Thus, in vivo subthreshold membrane potentials are underestimated by ∼10% (e.g., a measured −50 mV corresponds to a true value near −55 mV). Recordings were performed in current-clamp configuration using an EPC-10 USB patch-clamp amplifier (HEKA Elektronik). The data were acquired with the PatchMaster software (HEKA Elektronik; RRID:SCR_000034) at a 20 kHz sampling rate and filtered with a 5 kHz Bessel low-pass filter.

### In vivo patch-clamp recordings

In vivo patch-clamp recordings were performed as previously described (Otomo et al., 2020). Patch pipettes (8–12 MΩ) were pulled from borosilicate glass capillaries (G120F-4, Warner Instruments) using a horizontal pipette puller (DMZ-Universal-Electrode-Puller, Zeitz-Instruments). Patch pipettes were filled with the same internal solution used for in vitro patch-clamp recordings. During recording, animals were anesthetized using isoflurane (1.0–2.5% in O_2_, 0.35 L/min) and fixed with a head-plate to a customized recording platform (Luigs & Neumann). Throughout the recording, body temperature (37–38°C) and respiratory rate (1–2 Hz) were regularly monitored and adjusted when necessary. Craniotomy was performed at a previously made reference location. High positive pressure (600–1,000 mbar) was maintained during lowering of the electrode to ∼200 µm above the region of interest. The pressure was gradually lowered (40–70 mbar) during probing for neurons within the area of SN (DV, 3.8–5.2 mm). A sound generating device (PSA-12, HEKA Elektronik) was used for continuous monitoring of the pipette resistance during probing and sealing. A fluctuating increase (20–50%) of the resistance indicated the proximity of the plasma membrane to the electrode tip. Once whole-cell configuration was obtained, cell capacitance and series resistance were estimated by the recording software (PatchMaster, HEKA Elektronik; RRID:SCR_000034). The data were acquired using a patch-clamp amplifier (EPC-10 USB, HEKA Elektronik) at a sampling rate of 20 kHz and filtered with a low-pass filter (Bessel, 5 kHz). Whole-cell recordings of spontaneous neuronal activity were obtained using current-clamp configuration with zero holding current (*I* = 0). Hyperpolarizing current injections were used to evoke subthreshold responses including sag and rebound firing.

### Immunohistochemistry and anatomical localization of in vitro and in vivo recorded neurons

Immunohistochemistry and confocal microscopy were performed as previously described (Farassat et al., 2019; Otomo et al., 2020; Shin et al., 2022). For in vitro patch-clamp experiments, midbrain sections containing recorded and NB-filled cells as well as forebrain tissue blocks with striatal injection sites were transferred to fixative [4% paraformaldehyde (PFA) and 15% picric acid in phosphate-buffered saline (PBS) solution], pH 7.4, and kept overnight at 4°C for postfixation. On the following day, the tissue was transferred to a 10% sucrose/0.05% NaN_3_ solution for long-term storage. Striatal injection sites were sectioned into serial 100 µm coronal slices using a vibrating blade microtome (VT1000S, Leica Biosystems). On the first day, striatal sections or midbrain slices were rinsed in PBS and then incubated with blocking solution (10% horse serum, 0.5% Triton X-100, 0.2% bovine serum albumin in PBS) for 2 h at room temperature. Afterward, sections were incubated with carrier solution (1% horse serum, 0.5% Triton X-100, 0.2% bovine serum albumin in PBS) containing the primary antibody overnight at room temperature. On the second day, sections were again rinsed three times in PBS and incubated (at room temperature, overnight) with the secondary antibody. For identifying NB-filled cells, midbrain sections were additionally incubated with streptavidin (1:750, Streptavidin Alexa Fluor 405, Invitrogen, #S32351). On the third day, sections were washed in PBS and mounted on slides with antifade mounting medium (VECTASHIELD, Vector Laboratories). Following in vivo patch-clamp recordings, mice were terminally anesthetized using pentobarbital (0.3–0.4 ml, Narcoren, Boehringer Ingelheim) and transcardially perfused with a fixative (4% PFA and 15% picric acid in PBS). Fixed brains were sectioned into serial 60 µm coronal slices using a vibrating blade microtome (VT1000S, Leica Biosystems). Further immunohistochemical processing was carried out as described above. For identifying NB-filled cells streptavidin conjugate (1:750, Streptavidin Alexa Fluor 568, Invitrogen, #S11226) was used. For a complete list of primary and secondary antibodies, see Table 1.

**Table 1.** Antibodies

Antibody	Species	Supplier, ID, RRID	Dilution	Experiment^a^
Anti-tyrosine hydroxylase	Rabbit, polyclonal	Millipore	1:1,000	1, 2, 3, 4
		catalog # 657012 RRID:AB_2313810		
Anti-tyrosine hydroxylase	Mouse, monoclonal	Millipore catalog #MAB318 RRID:AB_2201528	1:1,000	4
Anti-Aldh1a1	Goat, polyclonal	R&D Systems catalog #AF5869 RRID:AB_2044597	1:1,000	4
Anti-Anxa1	Rabbit, polyclonal	Innovative Research catalog #71-3400 RRID:AB_88080	1:1,000	4
Anti-calbindin	Mouse, monoclonal	Swant catalog #CB300PUR RRID:AB_3542811	1:1,000	4
Anti-rabbit Alexa Fluor 488	Goat, polyclonal	Thermo Fisher Scientific catalog #A-11008 RRID:AB_143165	1:750	1, 2, 3, 4
Anti-rabbit Alexa Fluor 405	Goat, polyclonal	Molecular Probes catalog #A-31556 RRID:AB_221605	1:750	4
Anti-rabbit Alexa Fluor 405	Donkey, polyclonal	Thermo Fisher Scientific catalog #A48258 RRID:AB_2890547	1:750	4
Anti-mouse Alexa Fluor 405	Goat, polyclonal	Thermo Fisher Scientific catalog #A-31553, RRID:AB_221604	1:750	4
Anti-mouse Alexa Fluor 488	Goat, polyclonal	Thermo Fisher Scientific catalog #A-11001 RRID:AB_2534069	1:750	4
Anti-goat Alexa Fluor 488	Donkey, polyclonal	Thermo Fisher Scientific catalog #A-11055, RRID:AB_2534102	1:750	4

a 1, Immunohistochemistry and anatomical localization of in vitro recorded neurons. 2, Immunohistochemistry and anatomical localization of in vivo recorded neurons. 3, Mapping of striatal injection sites. 4, Quantification of molecular markers in projection-defined midbrain DA neuron subpopulations.

### Confocal imaging

Confocal images were taken using a laser-scanning microscope (Nikon Eclipse90i, Nikon). Images were acquired and exported using NIS-Elements C program (Nikon software; RRID: SCR_014329). Low-magnification overview images of the striatal injection sites were taken with a 4× objective. Overview images of the midbrain section were acquired using a 20× objective. Images of the recorded and NB-filled cells were acquired with a 60× oil immersion objective.

### Quantification of molecular markers in projection-defined midbrain DA neuron subpopulations

To quantify expression of distinct molecular markers (Aldh1a1, Anxa1, and Calb1) within projection-defined DA subtypes in the midbrain, we infused three animals in each group (DLS, DMS, and lNAcc) with RB and processed after a survival period necessary for sufficient labeling. Subsequent tissue fixation and histological processing were carried out as described above. Three sets of alternating 30 µm midbrain sections (AP, −2.70 to −4.04 mm) from each animal were collected and immunohistochemically stained for the three molecular markers in combination with a TH staining to identify DA neurons. For a list of primary and secondary antibodies used, see Table 1. Low-magnification images of the sections were acquired with a 4× objective. Sections were defined using neuroanatomical landmarks and labeled according to their distance relative to the bregma based on a mouse brain atlas (Paxinos and Franklin, 2012). We used 20× z-stack images acquired with 5 µm steps for cell counting. RB- and TH-double positive as well as RB-, TH-, and Aldh1a1/Anxa1/Calb1-triple positive neurons were counted manually using cell counter plugin in ImageJ (RRID:SCR_003070). High-magnification images of retrogradely traced neurons were acquired using a 60× oil immersion objective.

### Confocal imaging and mapping of RB injection sites

For identification of local RB injection sites, all forebrain sections containing the RB signal were analyzed. Sections were defined according to their distance relative to the bregma using neuroanatomical landmarks and further aligned to their corresponding schematic coronal drawing from the mouse brain atlas (Paxinos and Franklin, 2012). The boundaries of tracer injection sites in individual animals were mapped manually using Adobe Illustrator (RRID:SCR_010279).

### Data analysis

In vitro and in vivo patch-clamp data were digitally filtered at 1 kHz and further exported as MAT-files using Fitmaster (HEKA Elektronik, RRID:SCR_016233). Offline data analysis was performed using custom-written scripts in MATLAB (R2022b, MathWorks; RRID:SCR_001622). For in vitro data, spike thresholds were determined using the first derivative of the recording where d*V_m_*/d*t* ≥ 10 mV/ms. For direct comparison of sag amplitudes between different DA neurons, the amplitudes of the negative current injections were adjusted for each cell individually to result in a peak hyperpolarization to −80 mV. Sag amplitudes were determined as repolarization from −80 mV to a steady-state value during the 2 s current injection protocol. Rebound delays were determined as the time between the end of the hyperpolarizing current injection and the peak of the first rebound action potential. Input resistances were calculated from the slope of the voltage–current relationship obtained from voltage responses to multiple steps of hyperpolarizing current injections. For in vivo data, detection of spike threshold was done as previously described (Otomo et al., 2020). A spike was detected if the following three conditions were met within a 3 ms time frame: First, for crossing the threshold, d*V_m_*/d*t* ≥ 10 mV/ms must be reached. Second, the minimal value of d*V_m_*/d*t* must be <−5 mV/ms. Third, the maximal value of the voltage must be >10 mV of the mean voltage in the entire trace. Spike thresholds, peaks, and minima after hyperpolarization potential were visually verified for each cell. For detection of spikes fired in burst, criteria previously described in Grace and Bunney (1984) were applied, where the burst onset is defined as the concurrence of two spikes with an interspike interval (ISI) of <80 ms and burst termination where an ISI exceeds value of >160 ms. For quantification of other values (sag amplitudes, input resistances, rebound delays), the same criteria as for in vitro data were applied.

### Drugs

All drugs were prepared as stock solutions and were stored at −21°C before use. Most drugs were water soluble; however, stock solution of isradipine was made in dimethyl sulfoxide (DMSO, Sigma-Aldrich). The final content of DMSO in extracellular solution did not exceed 0.1%. All drugs were applied via the superfusion of extracellular solution for ∼20 min before recordings were started. For a complete list of the drugs used, see Table 2.

**Table 2.** Drugs

Chemical	Bath conc.	Source	Identifier
AmmTx3	1 µm	Alomone Labs	Catalog #STA-305
Apamin	300 nm	Tocris Bioscience	Catalog #1652
CGP 55845	50 nm	Tocris Bioscience	Catalog #1248/10
CNQX	20 µm	Tocris Bioscience	Catalog #1045/1
DL-AP5	10 µm	Tocris Bioscience	Catalog #0105/10
Isradipine	300 nm	Tocris Bioscience	Catalog #2004/10
Ivabradine	25 µm	Tocris Bioscience	Catalog #6542
NNC 55-0396	70 µm	Tocris Bioscience	Catalog #2268
Oxotremorine-M	1 µm	Tocris Bioscience	Catalog #1067/100
Phenylephrine	10 µm	Tocris Bioscience	Catalog #2838/100
(S)-(-)-Sulpiride	600 nm	Tocris Bioscience	Catalog #0895
SR95531	4 µm	Tocris Bioscience	Catalog #1262/10
Tertiapin-Q	1 µm	Alomone Labs	Catalog #STT-170

### Computational model construction

This multicompartmental model (Amini et al., 1999; Kuznetsova et al., 2010) consists of a soma with two primary dendritic branches that each split into two distal branches 75–150 µm from the soma. From one of the primary dendrites originates an additional 50-µm-long dendritic branch 50 µm from the soma. This (along with the primary dendrite between it and the soma) represents the axon-bearing dendrite (ABD) is connected to the axon initial segment (AIS), consistent with DA SN neurons (Meza et al., 2018). The dendrites are further electrically segregated into 114 segments for accurate computation under both low- and high-frequency events. The axial resistance (200 Ω/cm) was chosen based on measurements of the length constant in simultaneous dendritic and somatic recordings (Häusser et al., 1995). To account for variation within identified subpopulations and decrease the likelihood that results correspond to overfitting, parameter ranges for each projection-specific DA model by parameter sweeps (Goldman et al., 2001). Individual model cells were then generated by random sampling from those parameter ranges using independent uniform distributions. The parameter ranges for the three different DA models are given in Table 3. The spiking conductances consist of a reduced Markov-based sodium channel (Balbi et al., 2017; Knowlton et al., 2021) and delayed rectifier potassium channel to produce action potentials, with a fivefold increase in conductance in the AIS compartment and decreased conductance in distal compartments, consistent with a drop in the action potential size with distance from the soma (Häusser et al., 1995). Spiking channel conductances were tuned to produce action potentials with peak voltages between +10 and +30 mV with an AIS-initiated spike with a threshold close to −40 mV and a width of 2 ms (at threshold) consistent with conventional DA SN neurons (Lammel et al., 2008). The model also makes subtype-specific assumptions on functional coupling between Ca_v_ channels and SK channels. CaL channels were found to amplify firing frequencies in DLS-projecting cells (Shin et al., 2022) indicating reduced coupling between CaL and SK channels (i.e., larger Ca^2+^ microdomain size in the model). Thus, the model assumes that Ca_v_1.3 is sufficiently segregated from SK channels for the channel to function in a purely electrogenic manner. While the coupling between SK and Ca_v_3 in postnatal DA SN neurons was previously shown (Wolfart and Roeper, 2002), the timescale and scope of this coupling in adult DA SN neurons was unknown. Therefore, the model assumed that a fraction of Ca_v_3 channels (Fig. 7*E*, f) is coupled to SK channels with the timescale depending on the size of the Ca_v_3 Ca^2+^ microdomain. Parameters were refined by iterative sweeps to best describe the experimental responses to selective ion channel blockers. For equations used in the model, see Text S1.

**Table 3.** Model parameters

Parameter	DLS-DA	DMS-DA	lNAcc-DA
gNa_L_ (µS/cm^2^)^a^	3.5–4.5	4–5	4–5
gK_L_ (µS/cm^2^)^a^	6–7	6–7	6–7
tau K_v_4 (ms)	25–50	75–125	60–100
gK_v_4 (µS/cm^2^)^b^	D: 135–225	D: 225–360	D: 225–360
	S: 675–1,125	S: 1,125–1,800	S: 1,125–1,800
gCaT (µS/cm^2^)^b^	<150^c^: 0	<150^c^: 0	<150^c^: 0
	>150^c^: 350–450	>150^c^: 150–250	>150^c^: 150–250
gSK (µS/cm^2^)	75–150	100–200	100–200
gHCN (µS/cm^2^)	15–20	15–20	15–20
gGIRK (µS/cm^2^)	20 (in vitro)	20 (in vitro)	20 (in vitro)
	10–30 (in vivo)	10–30 (in vivo)	10–30 (in vivo)

a Sodium and potassium leak conductance are varied in tandem such that their sum is unchanged.

b Parameters with differing values with distance from soma maintain a fixed ratio between distances.

c Distance from the soma in micrometer. D, dendrite; S, soma.

### Placing the projection-specific DA SN models in a simulated in vivo environment

Simulated in vivo environments were generated through the application of Poisson distributed glutamate synaptic inputs, activating both AMPA and NMDA receptors, as well as independent Poisson distributed inhibitory GABA_A_ synaptic inputs. The synaptic contacts were assumed to be distributed uniformly over the dendritic tree, with each synaptic event being assigned to a given compartment (except for the AIS) with a probability proportional to that compartment's surface area. D2 and GABA_B_ receptor-mediated GIRK activation was assumed to be driven by a basal time-invariant GABA and DA tone. The level of synaptic inputs was chosen such that the in vivo mean frequency remained in the experimentally observed 1–8 Hz frequency rage while simultaneously generating about twofold reduction input resistance along with an increase in firing variability (CV) that matched experimental in vivo results (Farassat et al., 2019). Simulated in vivo experiments were performed in two ways. The first, replicating a typical in vivo recording of a DA cell, uses an individual cell with repeated trials, each with a separate representation of the balanced state noise to isolate the effects of the noise from the intrinsic dynamics. The second, representing the predicted response of a population of cells to correlated inputs, generated 30 representations of the randomized model parameters for each of the specific projections. Each cell was then presented with a single hyperpolarizing step, and statistics were generated from the resulting traces that produced both pre- and postrebound spiking. Simulations were performed in Python using the NEURON package (Hines and Carnevale, 1997; Hines et al., 2009).

### Statistical analysis

Statistical tests and graphs were made using GraphPad Prism 10 (RRID: SCR_002798). Initially, normality of all datasets was assessed using the single-sample Kolmogorov–Smirnov test. For datasets that followed a normal distribution, statistical significance was evaluated using an unpaired two-tailed Student's *t* test, one-way analyses of variance (ANOVA), and repeated-measures two–way ANOVA. Multiple-comparison corrections were applied post hoc using Tukey's, Bonferroni's, and Dunnett's tests. If datasets did not meet normality assumptions, statistical analyses were conducted using the two-tailed Mann–Whitney test or a mixed-effects model. The statistical significance level was set to *p* < 0.05 in all tests. The sample sizes, statistical tests applied, and main effects for each analysis are individually reported in the Results section.

### Code accessibility

The code used for computational modeling experiments is freely available on ModelDB (https://modeldb.science/2018020).

## Results

### Projection-defined subpopulations of midbrain DA neurons display distinct profiles of molecular markers

**Figure 1.**
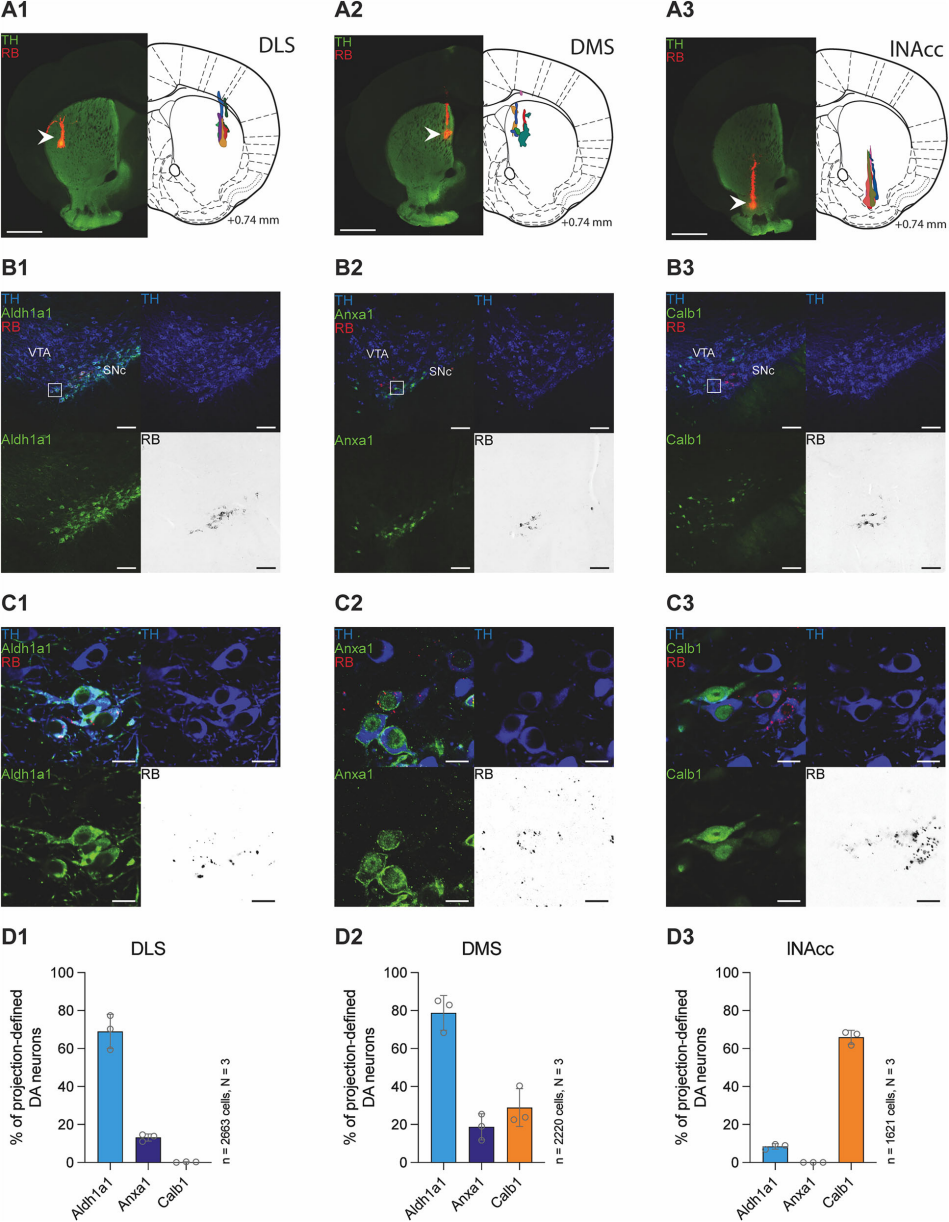
Molecular expression profiles of retrogradely traced subpopulations of DA SN neurons. ***A*1–3**, Injection sites for retrograde tracing experiments injecting fluorescently labeled red retrobeads into DLS (***A*1**; bregma +0.74 mm), DMS (***A*2**; bregma +0.74 mm), and lNAcc (***A*3**; bregma +0.74 mm). Left panels show confocal images of injection sites with merged TH (green) and RB (red) signal. Arrowheads indicate the respective injection sites with the reservoir of the RB tracer. Right panels show schematic drawings of the corresponding brain regions and outline manually mapped tracer areas from individual animals, marked with different colors. Scale bars, 1 mm. ***B*1–3**, Confocal 20× images of retrogradely RB-traced (red in merged image, black in isolated image) midbrain DA neurons (injection site, DMS) costained for TH (blue) and Aldh1a1 (***B*1**, green) or Anxa1 (***B*2**, green) or Calb1 (***B*3**, green). Scale bars, 100 µm. ***C*1**,**2**, Zoomed-in windowed areas shown in ***B*1–3**. Confocal images of retrogradely RB-traced (red in merged image, black in isolated image) midbrain DA neurons at higher magnification (60×), costained for TH (blue) and Aldh1a1 (***C*1**, green) or Anxa1 (***C*2**, green) or Calb1 (***C*3**, green). Scale bars, 15 µm. ***D*1–3**, Bar graphs showing average percentages of retrogradely RB-labeled midbrain DA neurons expressing Aldh1a1, Anxa1, or Calb1, respectively, across distinct projection sites (***D*1**, DLS; ***D*2**, DMS; ***D*3**, lNAcc; *N* = 3 mice per group). Note that DLS-projecting DA neurons lack Calb1 expression, while lNAcc-projecting DA neurons do not express Anxa1. Interestingly, midbrain DA neurons projecting to DMS express all three molecular markers, although to a variable degree.

Based on numerous studies that identified multiple subtypes of DA neurons with their different molecular properties (Poulin et al., 2014, 2018; Saunders et al., 2018) and our previous works (Lammel et al., 2008; Farassat et al., 2019) that demonstrated axonal-projection–related differences in electrophysiological properties of midbrain DA neurons in vitro and in vivo, we investigated molecular expression and electrophysiological profiles of distinct projection-defined midbrain DA subpopulations. To address this question, we combined axonal tracing experiments using RB with additional immunohistochemistry for three molecular markers (Aldh1a1, Anxa1, and Calb1). For retrograde labeling, we targeted all three axonal projections of DA SN neurons: DLS (*N* = 3 animals; Fig. 1*A*1), DMS (*N* = 3 animals; Fig. 1*A*2), and lNAcc (*N* = 3 animals; Fig. 1*A*3). Following retrograde labeling, we mapped the respective striatal injection sites and quantified the expression of molecular markers in TH+ (i.e., DA) neurons. Figure S1 documents the selectivity of all injection sites used for the anatomical mapping of all three different targets by their complete rostrocaudal serial reconstructions. The overall topographic pattern of distributions of TH+ neurons (Fig. S2) was in accordance with those from previous work using Green Retrobeads, FluoroGold, or retrograde viral tracers (Lammel et al., 2008; Lerner et al., 2015; Farassat et al., 2019). Furthermore, immunohistochemical staining was done on midbrain sections to examine the expression of molecular markers Aldh1a1, Anxa1, and Calb1 in retrogradely traced DA SN neurons (Fig. 1*B*1–*C*3).

Quantitative analysis revealed distinct expression patterns of these molecular markers among the different projection-defined subpopulations of midbrain DA neurons. DLS-projecting DA neurons (Fig. 1*D*1) predominantly expressed Aldh1a1 (mean, 69%; min, 59.38%; max, 77.37%) and Anxa1 (mean, 13.19%; min, 11.07%; max, 14.63%). However, close to none of DLS-projecting DA neurons expressed Calb1 (mean, 0.22%; min, 0%; max, 0.34%), which was well in accordance with the findings from our previous work (Farassat et al., 2019). Interestingly, DA neurons projecting to DMS showed a more heterogeneous expression profile (Fig. 1*D*2). These neurons expressed all three molecular markers—Aldh1a1, Anxa1, and Calb1—although to varying extents. Comparable with DLS-projecting neurons, ∼80 and 20% of DMS-projecting DA neurons expressed Aldh1a1 (mean, 78.77%; min, 68.25%; max, 85.13%) and Anxa1 (mean, 18.75%; min, 11.73%; max, 25.49%), respectively. In contrast to DLS-projecting, DMS-projecting SN DA neurons displayed more abundant Calb1 expression (mean, 28.92%; min, 22.56%; max, 40.40%).

This implies that DLS- and DMS-projecting DA SN neurons cannot be well segregated by the panel of the molecular markers used here. In contrast, lNAcc-projecting DA SN neurons were easy to differentiate from dorsal striatum-projecting DA SN neurons by their abundant Calb expression (mean, 65.94%; min, 61.73%; max, 68.45%) and the absence of Aldh1a1 and Anxa1 (Aldh1a1, mean, 8.47%; min, 6.87%; max, 9.55%; Anxa1, mean, 0%; min, 0%; max, 0%).

### DLS-projecting SN DA neurons display unique properties of rebound excitability

**Figure 2.**
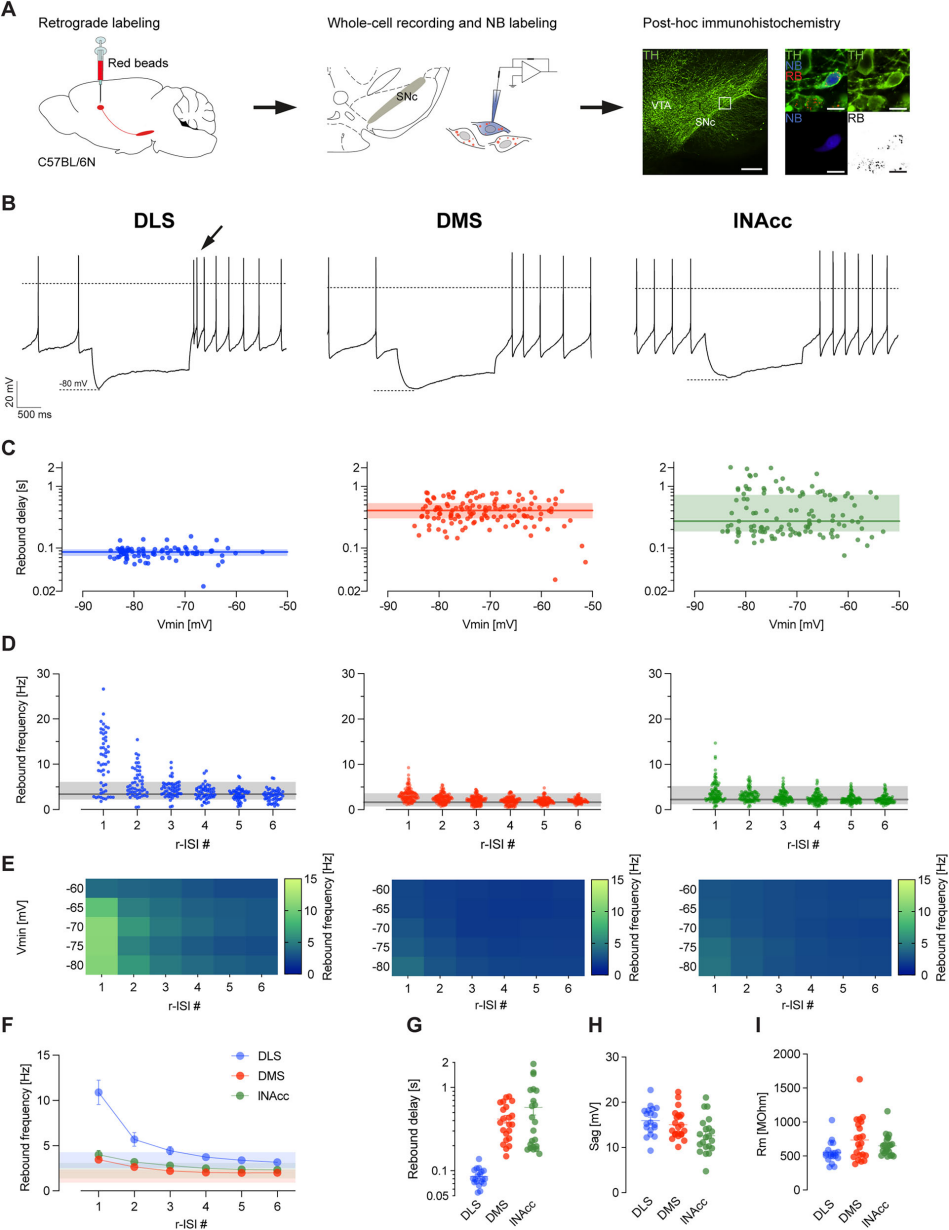
Synaptically isolated DLS-projecting DA SN neurons display unique high rebound frequencies. ***A***, Left, Schematic illustration of a sagittal section of a mouse brain. RB were injected into DLS, DMS, and lNAcc to retrogradely label DA SN neurons. Middle, During whole-cell recordings, neurons were filled with 0.2% NB for post hoc identification. Right, Lo-magnification (20×) confocal image of rostral midbrain section stained for TH (green, left panel). White rectangle represents the area shown in the high-magnification images. High-magnification (60×) confocal images showing NB-labeled (blue, right-bottom left panel), TH-positive (green, right-top right panel), and RB-containing (black, right-bottom right panel) DA SN neuron. Scale bars, 100 and 15 µm. ***B***, Representative examples of current-clamp recordings of retrogradely labeled and synaptically isolated DA SN neurons with membrane voltage responses to 2 s injections of negative current to hyperpolarize the cell initially to −80 mV (including inhibition of AMPAR, NMDAR, GABA_A_, GABA_B_, and D2R). Note the presence of rebound bursts in DLS-projecting DA SN neurons (arrow). Dashed lines indicate membrane potential at 0 mV (top line) and −80 mV (bottom line). Scale bars, 20 mV and 500 ms. ***C***, Log-scale scatterplot showing the distribution of rebound delay responses and corresponding minimal membrane voltage (*V*_min_) during 2 s negative current injection. Each dot represents an individual recording trace. The colored horizontal line represents the median; the shaded area represents the interquartile range. Note that DLS-projecting DA SN neurons display a compressed dynamic range of fast rebound delays compared with DMS- and lNAcc-projecting DA SN neurons. ***D***, A scatterplot showing rebound frequency of the first six rebound r-ISI # occurring after the end of current injection. Each dot represents an individual recording. The black line represents the mean; the shaded area represents the range of the baseline firing rate. Note that rebound frequency of DLS-projecting DA SN neurons strongly exceeds the baseline firing rate. ***E***, Heat maps illustrating the relationship between minimal membrane voltages (*V*_min_) during 2 s negative current injections and mean rebound frequencies for each of the six r-ISIs occurring after the end of current injection. For panels ***B–E***, data represent DA SN neurons projecting to the DLS, DMS, and lNAcc from the left to the right, respectively. ***F***, Plot showing the mean rebound frequency against the r-ISI number for DLS- (blue), DMS- (red), and lNAcc (green)-projecting DA SN neurons. Mean values are derived from the data presented in panel ***D***. Data are shown as mean ± SEM. Shaded areas represent mean ± SD of the baseline firing rate. Note that rebound frequency of DLS-projecting SN DA neurons highly exceeds baseline firing rates, while DMS- and lNAcc-projecting neurons display both rebound frequencies that stay within the baseline firing range. ***G–I***, Scatterplots of rebound delay (***G***) and sag amplitudes measured at *V*_min_ = −80 mV (***H***) and input resistances (***I***) for all recorded neurons. Horizontal lines represent mean ± SEM.

To address the question whether these DA SN subpopulations—as defined above—possess distinct electrophysiological properties, we combined retrograde labeling with whole-cell patch–clamp recordings in in vitro brain slices from 8- to 12-week-old adult C57Bl/6N mice. All in vitro recorded neurons reported in this study were labeled using NB for post hoc confirmation of the dopaminergic phenotype with identification of their respective axonal projection site, indicated by the RB signal within the TH-labeled cell (Fig. 2*A*).

Figure 2*B* shows representative examples of current-clamp recordings of retrogradely labeled DA neurons projecting to DLS, DMS, and lNAcc in response to 2 s negative current pulses (scaled to reach a maximal hyperpolarization of about −80 mV). Note the significantly shorter rebound delay and higher rebound frequency in DLS-projecting DA neurons (see arrow). In other words, only DLS-projecting DA SN neurons displayed intrinsic rebound bursting, like thalamic neurons (McCormick and Prince, 1986; Pape and McCormick, 1995). In contrast, DMS- and lNAcc-projecting DA SN neurons displayed slow, ramp-like depolarization of the membrane potential after a termination of the hyperpolarizing current pulse. This rebound burst phenotype has been previously described, but not yet associated with the particular axonal projection (Brischoux et al., 2009; Wang and Tsien, 2011; Fiorillo et al., 2013; Evans et al., 2017, 2020). To quantify rebound properties of the three projection-defined DA SN neurons, we plotted rebound delays against the maximal hyperpolarization induced by current injection (Fig. 2*C*). In the subthreshold range between −55 and −85 mV, the rebound delays were significantly different between the three projections. However, all three projections were independent of the hyperpolarization amplitude within the subthreshold range (DLS, mean, 0.086 s; DMS, mean, 0.427 s; lNAcc, mean, 0.480 s; DLS vs DMS vs lNAcc, mixed-effects model, Tukey's multiple-comparison test; *p* < 0.0001).

To reveal the details of rebound activity, we plotted the first six rebound ISIs (r-ISI; Fig. 2*D*) with reference to the cells’ baseline activity (Fig. 2*D*, gray bars; DLS, mean, 3.4 Hz; SD, 0.88; DMS, mean, 1.63 Hz; SD, 0.72; lNAcc, mean, 2.24 Hz; SD, 0.85). We found only for DLS-projecting cells that the first two r-ISIs were clearly outside of the spontaneous pacemaker range (r-ISI 1, 1.8–26.6 Hz; r-ISI 2, 0.5–15.43 Hz; baseline frequency range, 2.2–6.1 Hz; Kruskal–Wallis one-way ANOVA test; *p* < 0.0001). Note that we observed a gradient of rebound excitability among DLS-projecting DA SN neurons with some cells showing high rebound excitability (>15 Hz), while others remained within the pacemaker range. In contrast, DMS- and lNAcc-projecting neurons displayed a more compressed rebound excitability with most cells firing within the pacemaker range (DMS r-ISI 1, 1.11–9.28 Hz; baseline frequency range, 0.7–3.6 Hz; lNAcc r-ISI 1, 0.44–14.7 Hz; baseline frequency range, 1.2–5.2 Hz). In summary, only DLS-projecting DA SN neurons possessed short and fast rebound bursts.

As shown in Figure 2*E*, we explored the voltage dependence of rebound bursting. The heat maps of the rebound frequency show robust rebound bursting in DLS-projecting DA SN neurons below −65 mV. In contrast, rebound bursting was not evoked in the other two projections across the entire subthreshold range. This demonstrates a fixed timing and gain of rebound bursts in a subset of DLS-DA neurons [rebound delay at −80 mV; (DLS) mean, 0.085 s; SD, 0.02; (DMS) mean, 0.42 s; SD, 0.199; (lNAcc) mean, 0.577 s; SD, 0.51, Kruskal–Wallis one-way ANOVA test; *p* < 0.0001; Fig. 2*F,G*].

In contrast to rebound properties, we found only small differences for other intrinsic properties, such as sag amplitude at −80 mV, and input resistance (Fig. 2*H*,*I*).

We further explored the time dependence of rebound bursting in DLS-DA by changing the duration of the preceding hyperpolarization from 100 to 2,000 ms (Fig. S3). We found that both rebound delay and frequency scale with the duration of hyperpolarization in an exponential fashion.

### SK channels prominently control the gain of rebound excitability in DLS-DA neurons

Next, we aimed to define the biophysical mechanism controlling selective rebound bursting in DLS-DA neurons. Several ion channels including, e.g., T-type Ca^2+^, small conductance Ca^2+^-activated K^+^ (SK), K_v_4, and hyperpolarization-activated cyclic nucleotide–gated (HCN) channels have been implicated in driving and controlling rebound bursting (Neuhoff et al., 2002; Amendola et al., 2012; Evans et al., 2017; Tarfa et al., 2017). For DA neurons, Evans and colleagues have recently shown that T-type Ca^2+^ channels are necessary for rebound excitability in a subpopulation of SN DA neurons (Evans et al., 2017, 2020).

**Figure 3.**
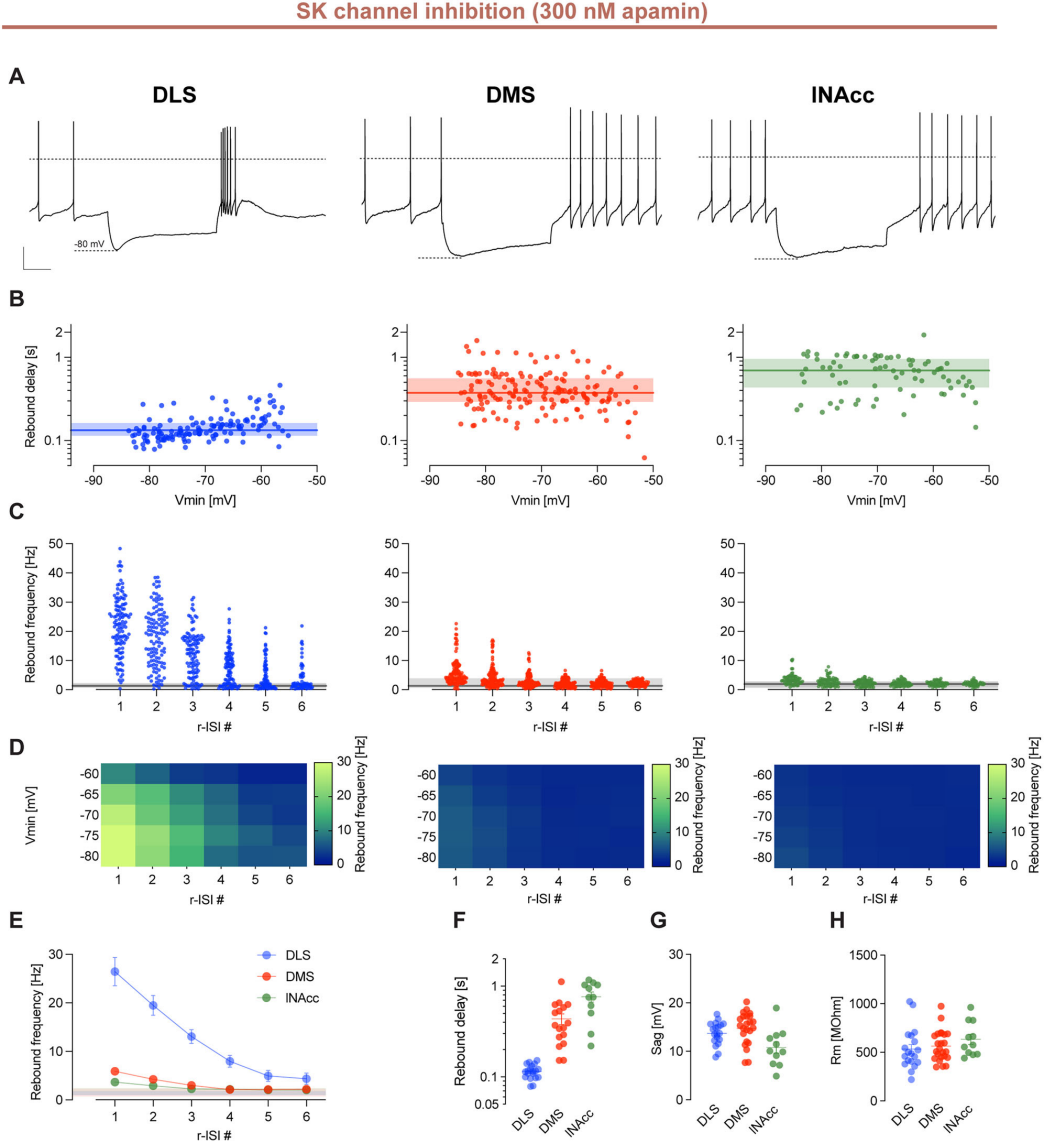
SK channel inhibition amplifies phenotypical differences among projection-defined subpopulations of DA SN neurons. ***A***, Representative examples of current-clamp recordings of retrogradely labeled DA SN neurons with membrane voltage responses to 2 s injections of negative current to hyperpolarize the cell initially to −80 mV in the presence of 300 nm apamin, a potent and highly selective inhibitor of SK channels. Dashed lines indicate membrane potential at 0 mV. Scale bars, 20 mV and 500 ms. ***B***, A log-scale scatterplot as in Figure 2*C*. Note that DLS-projecting DA SN neurons display a compressed dynamic range of fast rebound delays compared with DMS- and lNAcc-projecting SN DA neurons, also in the presence of 300 nm apamin. ***C***, Scatterplot as in Figure 2*D*. Note that inhibition of SK channels using 300 nm apamin leads to a strong increase in rebound frequency of DLS-projecting DA neurons. ***D***, Heat maps as in Figure 2*E*. For panels ***A–D***, data represent DA SN neurons projecting to the DLS, DMS, and lNAcc from left to right, respectively. ***E***, Plot as in Figure 2*F*. Shaded areas represent mean ± SD of the baseline firing rate in the presence of 300 nm apamin. Note that SK channel inhibition strongly affects DLS-projecting DA neurons, while DMS- and lNAcc-projecting DA neurons remain mainly unaffected. ***F–H***, Scatterplots as in Figure 2*G–I*.

First, we explored the potential role of SK channels that we have previously shown to control burst firing in neonatal DA SN neurons (Wolfart and Roeper, 2002). Preincubation with the selective SK channel inhibitor apamin (300 nm) extended the frequency range of rebound excitability in DLS-DA neurons about twofold, leading to rebound burst frequencies up to ∼50 Hz, with only small changes in DMS- and lNAcc-DA populations [r-ISI 1 (hertz), DLS, mean, 26.43; SD, 17.84; DMS, mean, 5.91; SD, 4.53; lNAcc, mean, 3.68; SD, 1.98; Fig. 3*A*–*C*,*F*, summarized in Fig. 3*E*,*F*]. In addition, apamin also prolonged rebound bursting in DLS-DA neurons about threefold (DLS, r-ISI 1–6 (hertz), mean, 26.43, 19.47, 13.07, 7.97, 4.96, 4.37; SD, 17.84, 12.25, 8.02, 6.53, 5.5, 5.04; Fig. 3*C*). In contrast, rebound delays were not affected [rebound delay (seconds); DLS, mean, 0.12; SD, 0.02; DMS, mean, 0.44; SD, 0.24; lNAcc, mean, 0.77; SD, 0.33; Kruskal–Wallis one-way ANOVA test; *p* > 0.05]. These data imply that SK channels control rebound gain (frequency and duration) without affecting rebound timing (Fig. 3*C*,*E*,*F*). Sag amplitudes and input resistances of all three populations were not affected by the presence of apamin (Fig. 3*G*,*H*).

As shown in Figure S4, apamin also decreased the duration of hyperpolarization necessary to evoke rebound bursting. In comparison to controls, rebound bursting already occurred consistently after 300 ms (after 1,000 ms in controls; compare Fig. S3).

In addition, we also explored the influence of intracellular calcium dynamics (Fig. S5; Table 4). Here we found that at the physiological intracellular calcium concentrations (estimated at ∼100 nm including the calcium buffering of K-gluconate), DLS-DA neurons displayed robust, fast rebound delays and high rebound frequency (Fig. S5*F*). While rebound bursting was dampened by increased intracellular calcium (∼350 nm in K-methanesulfonate internal solution), rebound bursting was enhanced by additional buffering of intracellular calcium via 1 mM EGTA or 1 mM BAPTA (Fig. S5*D*,*E*).

**Table 4.** Calcium concentrations

K-Anion	Ca^2+^ Buffer	Total Ca^2+^	Estimated free Ca^2+a^
KMeSO_3_	0.1 mM EGTA	0.075 mM	367.6 nm
K-Gluconate^b^	0.1 mM EGTA	0.075 mM	136.2 nm
K-Gluconate^b^	1 mM EGTA	15 µm^c^	0.74 nm
K-Gluconate^b^	1 mM BAPTA	15 µm^c^	0.74 nm

a Estimated free Ca^2+^ was calculated with the MaxChelator program used: experimental, two chelators, two metal calculators, constants from “Chelator” program; Link: https://somapp.ucdmc.ucdavis.edu/pharmacology/bers/maxchelator/downloads.htm.

b To account for the additional calcium buffering capacity of gluconate, all free Ca^2+^ concentrations calculated using MaxChelator were corrected by a factor of 2.7, as previously described by Woehler et al. (2014).

c No Ca^2+^ was added to the internal solution; however, a contamination level of 15 μm was assumed to account for calcium contamination typically present in K^+^-based solutions, as reported by Woehler et al. (2014).

### Inhibition of Ca_v_3 channel removes rebound bursting in DLS-DA neurons

**Figure 4.**
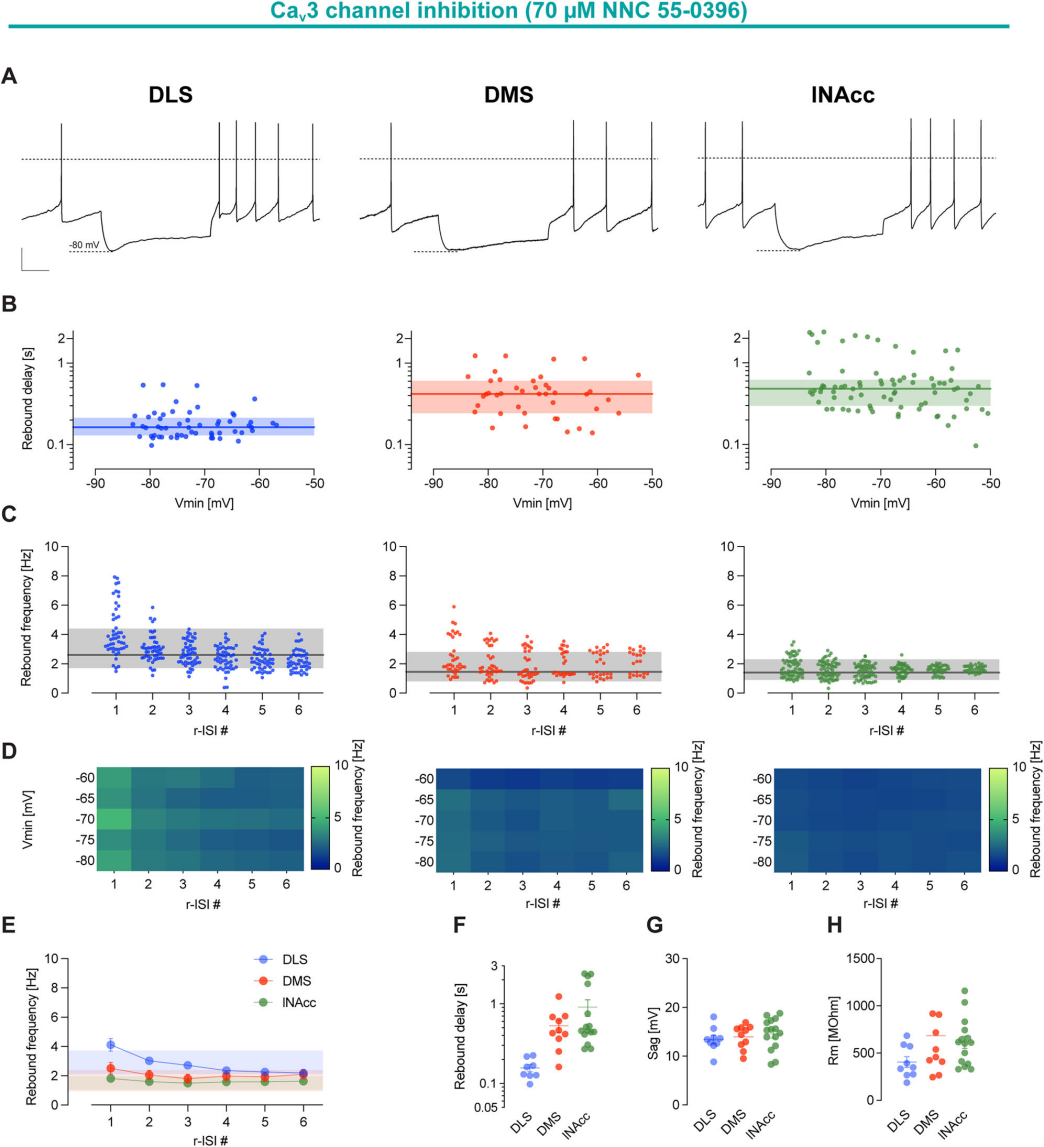
Inhibition of T-type Ca^2+^ channels reduce differences in rebound frequency between distinct projection-defined subpopulations of DA SN neurons. ***A***, Representative examples of current-clamp recordings of retrogradely labeled DA SN neurons with membrane voltage responses to 2 s injections of negative current to hyperpolarize the cells initially to −80 mV in the presence of 70 µm NNC 55-0396, a highly selective blocker of Ca_v_3 channels. Dashed lines indicate membrane potential at 0 mV. Scale bars, 20 mV and 500 ms. ***B***, A log-scale scatterplot as in Figure 2*C*. ***C***, Scatterplot as in Figure 2*D*. ***D***, Heat maps as in Figure 2*E*. For panels ***A–D***, data represent DA SN neurons projecting to DLS, DMS, or lNAcc from the left to the right, respectively. ***E***, Plots as in Figure 2*F*. Shaded areas represent mean ± SD of the baseline firing rate in the presence of 70 µm NNC 55-0396. ***F–H***, Scatterplots as in Figure 2*G–I*. Note that differences in rebound delays between distinct projection-specific DA SN subpopulations persist under inhibition of Ca_v_3 channel, while differences in rebound frequency are strongly reduced.

In accordance with Evans et al. (2017), inhibition of Ca_v_3 channels with 70 µm NNC 55-0396 almost completely removed rebound bursting in DLS-DA neurons (Fig. 4*A*–*D*). Maximal rebound frequencies were below 10 Hz, with prolonged rebound delays [DLS, rebound frequency r-ISI 1 (hertz), mean, 4.11; SD, 1.63; rebound delay (seconds), mean, 0.16; SD, 0.05; Fig. 4*E*,*F*]. In contrast, the properties of DMS- and lNAcc-DA neurons were only marginally affected [rebound frequency r-ISI 1 (hertz); DMS, mean, 2.51; SD, 1.29; lNAcc, mean, 1.82; SD, 0.69; rebound delay (seconds); DMS, mean, 0.53; SD, 0.30; lNAcc, mean, 0.91; SD, 0.82]. These results demonstrate a selective functional role of T-type Ca^2+^ channels in DLS-DA neurons.

Furthermore, T-type channels were necessary for the rebound excitability enhancing effect of apamin which was not affected by inhibition of L-type Ca^2+^ channels (Fig. S6). These data indicate a functional coupling between T-type Ca^2+^ channels and SK channels in DLS-DA neurons.

### K_v_4.3 channel inhibition shortens rebound delays in DLS-DA neurons

**Figure 5.**
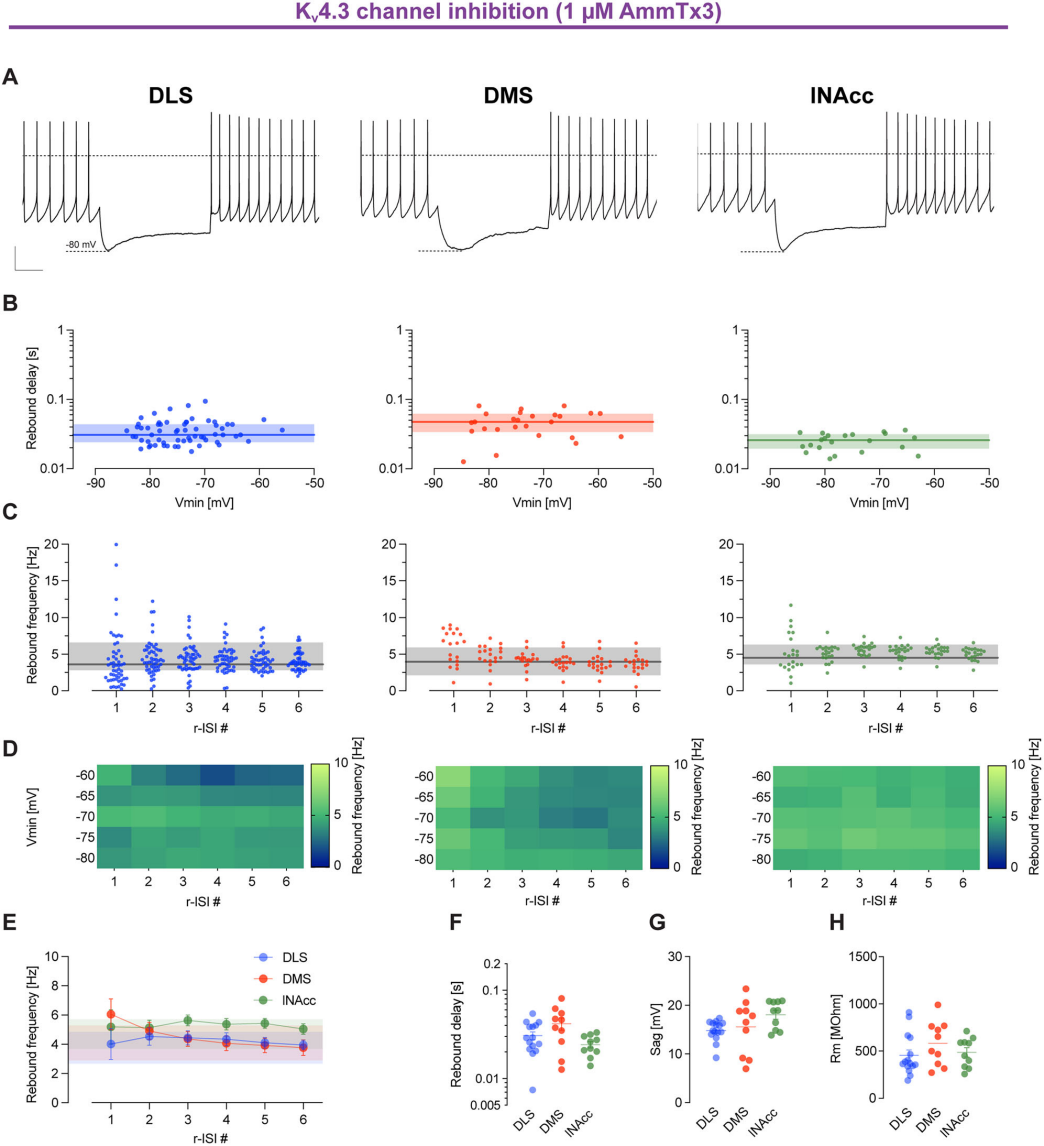
K_v_4.3 channel inhibition removes subthreshold differences among projection-defined subpopulations of DA SN neurons. ***A***, Representative examples of current-clamp recordings in retrogradely labeled DA SN neurons with membrane voltage response to 2 s injections of negative current to hyperpolarize the cells initially to −80 mV in the presence of 1 µm AmmTx3, a specific blocker of K_v_4 channel subunits. Dashed lines indicate membrane potential at 0 mV. Scale bars, 20 mV and 500 ms. ***B***, A log-scale scatterplot as in Figure 2*C*. ***C***, Scatterplot as in Figure 2*D*; the shaded area represents ±SD of the baseline firing rate. ***D***, Heat maps as in Figure 2*E*. For panels ***A–D***, data represent DA SN neurons projecting to the DLS, DMS, and lNAcc from the left to the right, respectively. ***E***, Plot as in Figure 2*F*. Shaded areas represent mean ± SD of the baseline firing rate in the presence of 1 µm AmmTx3. ***F–H***, Scatterplots as in Figure 2*G–I*. Note that the differences in rebound delay and rebound frequency across different projection-specific SN DA subpopulations under inhibition of K_v_4.3 channels are strongly reduced.

**Figure 6.**
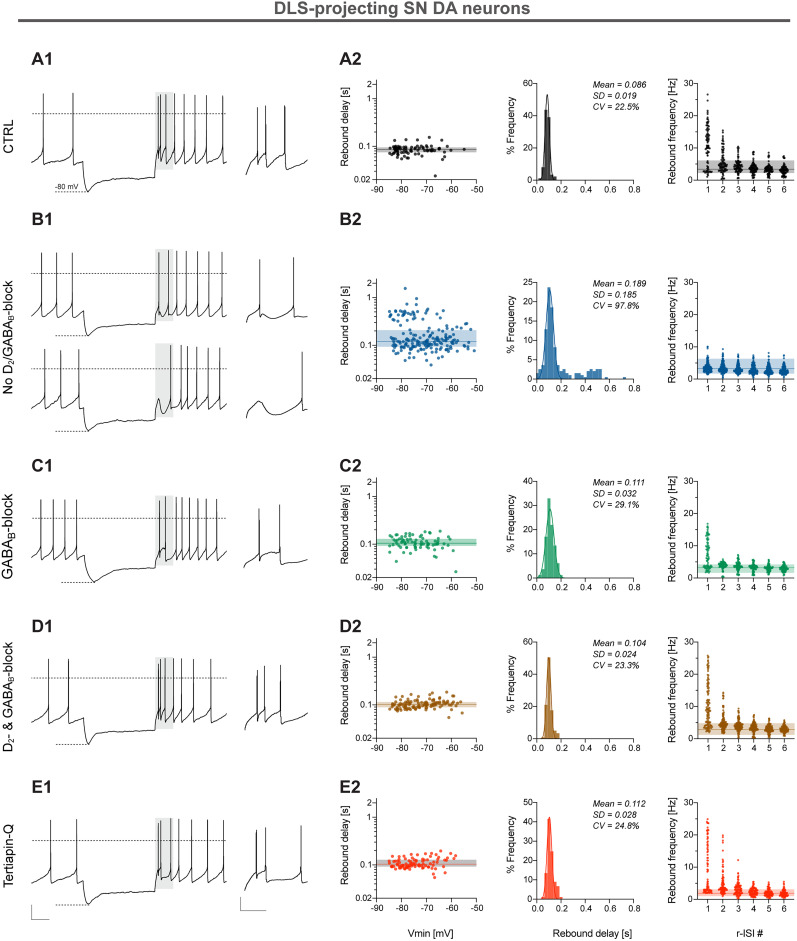
Rebound bursting of DLS-DA neurons is inhibited via G_αi_-mediated GIRK channel activity. ***A*1*–E*1**, Left, Representative traces of a current-clamp recording showing the rebound firing responses of retrogradely traced DLS-projecting DA SN neurons with membrane voltage response to 2 s injections of negative current under different recording conditions (indicated on the left). Right, Insets show a zoomed-in view of the windowed gray area. Dashed lines indicate membrane potential at 0 mV. Scale bars, 20 mV and 500 ms (left); 20 mV and 200 ms (right). ***A*2*–E*2**, Left, Log-scale scatterplots as in Figure 2*C* but showing the distribution of rebound delay responses and corresponding minimal membrane voltage (*V*_min_) during 2 s negative current injection. Middle, Distribution of rebound delay for each condition (colored lines, Gaussian fits). Right, Scatterplot as in Figure 2*F*.

Next, we studied K_v_4.3 channel inhibition using 1 µm AmmTx3 on rebound properties of DLS-DA neurons (Fig. 5*A*–*E*, compare with Fig. 6*B*). K_v_4.3 inhibition significantly reduced rebound delays in DLS-DA neurons to ∼30 ms [DLS, rebound delay (seconds); control, mean, 0.189; SD, 0.185; AmmTX3, mean, 0.03; SD, 0.01; compare Fig. 5*B*, left panel, with Fig. 6*B*]. However, rebound firing remained in the range of baseline firing frequencies under both conditions [DLS, control, rebound frequency r-ISI 1 (hertz), 3.54 ± 1.42; baseline (hertz), 3.22 ± 1.14; AmmTx3; rebound frequency r-ISI 1 (hertz), 4.02 ± 4.29; baseline (hertz), 3.75 ± 1.09; mean ± SD; compare Fig. 5*E* with Fig. 6*B*2]. We also noted that K_v_4.3 inhibition was most effective in removing the functional differences between the three projection-defined populations of DA SN neurons [rebound frequency r-ISI 1 (hertz); DMS, mean, 6.05; SD, 2.61; lNAcc, mean, 5.19; SD, 2.65; rebound delay (seconds); DMS, mean, 0.04; SD, 0.02; lNAcc, mean, 0.02; SD, 0.01; Fig. 5*E,F*]. This indicated that differences in K_v_4.3 channel gating and density are relevant to endow DA SN subpopulation with specific electrophysiological profiles.

We also explored the role of HCN channels for DLS- and DMS-projecting DA SN neurons (Fig. S7). Inhibition of these channels with 25 µm ivabradine effectively removed the sag component and increased the rebound delay in both DA subtypes. However, the gain of rebound bursting in DLS-DA neurons was not affected.

### GPCR-mediated GIRK channel activity dampens rebound bursting in DLS-DA neurons

As intrinsic excitability of DA subtypes is not only shaped by the expression and gating of voltage-gated channels but also controlled by neuromodulators, we next asked how rebound bursting is affected by D2 and GABA_B_ receptors, which both converge on a common pool of somatodendritic GIRK channels. In the absence of D2 and GABA_B_ blockers, we noted a large dispersion of rebound delays and a loss of rebound bursting [no D2/GABA_B_ block; rebound delay (seconds), mean, 0.189; SD, 0.182, CV, 97.84%; rebound frequency r-ISI 1 (hertz), mean, 3.54; SD, 1.42; Fig. 6*B*1,2] indicative of the presence of an endogenous tone of DA and GABA on these metabotropic GPCRs in adult mouse midbrain slices [rebound delay (seconds), GABA_B_ block; mean, 0.11; SD, 0.03; CV, 29.1%; D2 and GABA_B_ block; mean, 0.10; SD, 0.02; CV, 23.3%; rebound frequency r-ISI 1 (hertz), GABA_B_ block; mean, 7.43; SD, 4.3; D2 and GABA_B_ block; mean, 10.59, SD: 7.03; Fig. 6*A–D*]. Selective GIRK channel inhibition with 1 µm Tertiapin-Q was sufficient to reinstate rebound bursting similar to control levels which contained a cocktail of ionotropic and metabotropic inhibitors; see Materials and Methods [Tertiapin-Q, rebound delay (seconds), mean, 0.11; SD, 0.03; CV, 24.8%; rebound frequency r-ISI 1 (hertz), mean, 9.52; SD, 9.87; Fig. 6*E*1,2]. In summary, we show that G_αi_-mediated neuromodulation also controls rebound excitability in DLS-DA neurons. In contrast, we found no large effects of D2 and GABA_B_ receptor signaling on rebound properties of DMS- and lNAcc-projecting DA SN neurons (Fig. S8).

### Computational models of distinct DA SN subpopulations

**Figure 7.**
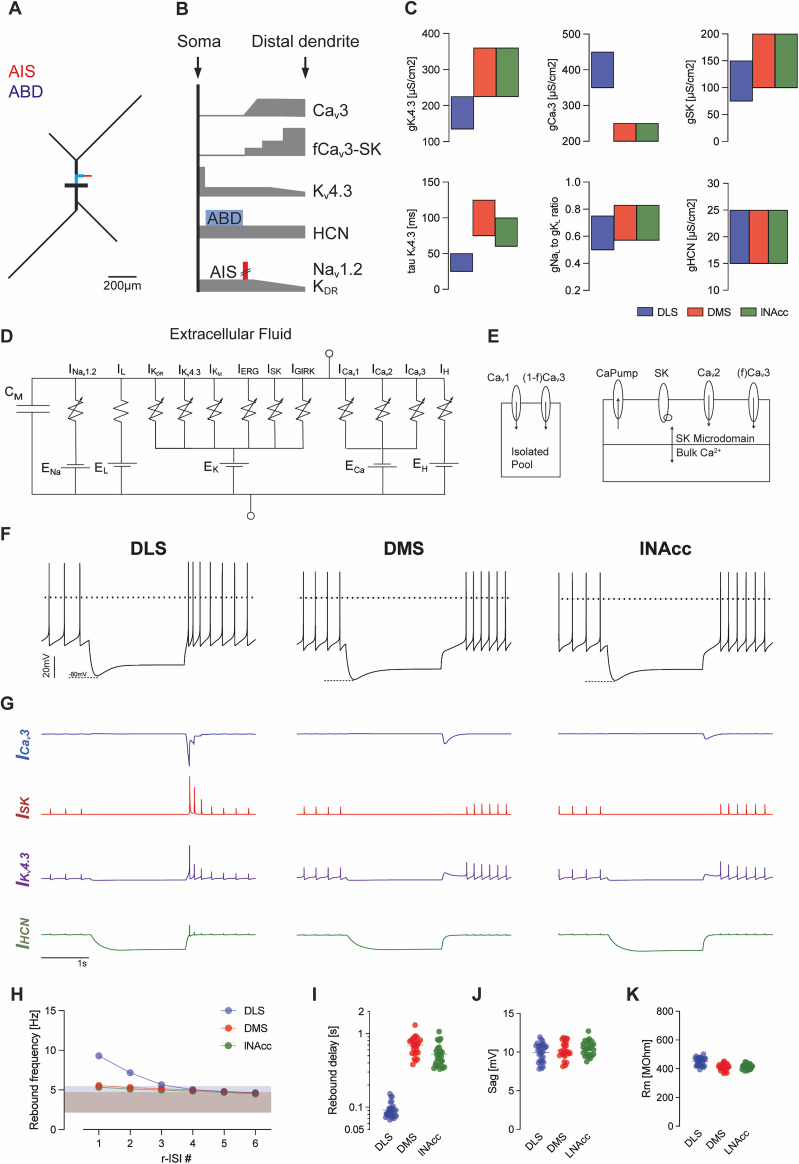
Computational models of morphologically realistic DA neurons with projection-specific parameters. ***A***, Schematic representation of multicompartmental model morphology with highlighted axon initial segment (AIS, red) and ABD (blue). Compartment diameter is exaggerated relative to length for clarity. ***B***, Spatial distribution of nonuniformly distributed ion channels along the somatodendritic tree. HCN channel density is higher at the ABD (blue rectangle) compared with the rest of the model. Na_v_1.2 and K_DR_ channel density is higher at the AIS (red rectangle) compared with the rest of the model. ***C***, Population-specific differences in model parameters. ***D***, Equivalent circuit diagram of a representative model compartment with an extracellular side of the model displayed on the top. ***E***, Schematic of calcium handling. (Right panel) SK channels are activated by a microdomain that senses both Ca^2+^ influx via the N-type Ca^2+^ channel (Ca_v_2) and a fraction (f) of the T-type (Ca_v_3) Ca^2+^ current. ***F***, Representative recording traces for each projection-defined SN DA model neuron with membrane voltage responses to simulated 2 s injection of negative current to hyperpolarize the model neurons initially to −80 mV. Horizontal dashed lines represent membrane voltage at 0 mV. ***G***, Summed currents of hyperpolarization-sensitive channels (from top to bottom, Ca_v_3 in blue, SK in red, K_v_4.3 in purple, and HCN in green) for representative model neurons. ***H***, Instantaneous frequencies (1/ISI) for the first six r-ISIs following simulated injection of hyperpolarizing current in which the minimum membrane potential during the step was −80 mV. Points/bars indicate mean ± SEM, shaded bands indicate mean ± SD of the baseline firing frequency for *N* = 11 randomized parameter sets for each subpopulation. Rebound delays (log scale, ***I***), sag potentials (***J***), and input resistances (***K***) of randomized models.

To gain more insight into the functional contributions of the experimentally characterized conductances, we constructed subtype-specific computational models. These model neurons used a common simplified morphology (Fig. 7*A*) consistent with previous projection-specific reconstructions (Hammer et al., 2024). Ionic channels and their kinetics were adapted from our previous single compartment model (Knowlton et al., 2021) with a focus on channels active during or recruited by hyperpolarization, including HCN (*I*_H_), Ca_v_3 (*I*_Ca,T_), and K_v_4.3 (*I*_A_) channels. The models also included voltage-gated Na^+^ (*I*_Na_) and delayed rectifier (*I*_K,DR_) channels for their role in the generation of action potentials. The currents mediated by Ca_v_2 (*I*_Ca,N_), SK (*I*_SK_), and K_v_7 (*I*_M_) were added for their role in the AHP (Shepard and Bunney, 1991; Drion et al., 2010; de Vrind et al., 2016). Ca_v_1.3 (*I*_Ca,L_) channels are also present for their role in amplifying pacemaker firing (Putzier et al., 2009; Philippart and Khaliq, 2018; Um et al., 2021; Shin et al., 2022; Cobb-Lewis et al., 2023). Finally, the model also contains K_v_11 (*I*_ERG_) channels necessary to maintain slow firing rates in the presence of SK channel blockers and terminate bursting in both simulated in vivo and in vitro environments (Canavier et al., 2007). GIRK (*I*_GIRK_) channels were included to model experiments shown in Figure 6.

Figure 7*B* shows schematically the spatial distribution of the relevant channels along the somatodendritic axis for all models. Acutely isolated DA neurons preserve only the dendrites within 50 μm of the soma, and Ca_v_3 is not observed in those preparations (Cardozo and Bean, 1995; Durante et al., 2004); therefore, we localized *I*_Ca,T_ in the more distal dendrites. We increased the transient outward conductance (K_v_4) in the soma compared with dendrites based on outside-out patch–clamp data showing that channel density of the transient outward current was greatest at somatic sites (Gentet and Williams, 2007). We also increased the hyperpolarization-activated conductance (g_H_) in the ABD according to the data from cell-attached patch–clamp recordings (Engel and Seutin, 2015). The fast voltage-gated sodium channel conductance (g_Na_) was highest in the AIS inferred from a study showing that the action potential is initiated at the AIS (Gentet and Williams, 2007) and lowest in the distal dendrites based on results obtained from localized application of TTX (Jang et al., 2014).

Model Figure 7*C* shows the different model parameter ranges for channel densities, channel gating kinetics, and Ca^2+^ microdomain sizes that emerged during projection-specific model development. Again, the differences were in part based on experimental data or were refined to describe projection-specific differences in the electrophysiological properties. For instance, as DLS-projecting neurons do not display pronounced ramp responses, their K_v_4.3 density was reduced. The DLS-projecting neurons were also assigned a faster time constant of inactivation of K_v_4 (τ_KA_) based on previous experimental literature (Kashiotis et al., 2011; Tarfa et al., 2017). Since ramp responses in DMS- and lNAcc-projecting neurons were removed by K_v_4.3 inhibition (Fig. 5), the gating parameters of Kv4.3 currents were tuned to reproduce those results. Low-voltage–activated (Ca_v_3) peak Ca^2+^ current amplitudes and current densities increased with a medial to lateral gradient for SN DA neurons. Lateral DA SN neurons mostly project to DLS (Farassat et al., 2019), so higher Ca_v_3 conductance densities were implemented in the model for DLS-projecting neurons. Finally, the SK conductance was smaller and with slower kinetics to reflect the reduced coupling between Ca^2+^ channels and SK channels in DLS-projecting DA SN neurons (Shin et al., 2022).

The equivalent circuit for a representative somatodendritic compartment of all models is shown in Figure 7*D* with the channels listed above in series with the membrane capacitance and a leak current (*I*_L_). For details of model development and refinement, see Materials and Methods.

Model Figure 7*E* provides a schematic view of the Ca^2+^ domains in the models. The calcium dynamics were implemented based on the constraints imposed by both previous studies and experimental results presented here. The AHP is driven primarily by coupling between the SK channels and conotoxin sensitive Ca_v_2 (N-type) channels, with no contribution of isradipine sensitive L-type Ca^2+^ channels (Ca_v_1.3; de Vrind et al., 2016). In the model, SK channels were activated by a microdomain (Fig. 7*C*) that senses both Ca^2+^ influx via the N-type Ca^2+^ channel and a fraction (f) of the T-type Ca^2+^ current.

Figure 7*F* shows a representative response of each modeled subpopulation to a square pulse of negative current with the parallel time-dependent changes of the model currents most closely related to rebound activity. The current amplitudes were titrated in each model to result in a maximal hyperpolarization to −80 mV. The values for these examples were randomly drawn from the model-specific parameter ranges. Similar to the experimental data (Fig. 2), only DLS-projecting DA SN model neurons responded with enhanced rebound excitability while the other two projection-specific models displayed pronounced ramp responses.

To test the robustness of differences between projection-specific DA SN models, we repeatedly chose random value sets from the respective model-specific parameter ranges (*n* = 11). Figure 7*G* plots the resulting rebound delays for the three model populations. Similar to the experimental data (Fig. 2*G*), the rebound delays of DLS-projecting DA SN model neurons are significantly shorter compared with the other two populations. Also, rebound frequency of DLS-projecting DA SN model neurons are significantly faster compared with the other two populations (Fig. 7*H*), consistent with the experimental data (Fig. 2*F*). These results demonstrate that the projection-specific models with their specific parameter ranges describe the experimental results under control conditions very well. In addition, we modeled the pharmacological experiments inhibiting in silico either SK, T-type Ca^2+^, or K_v_4.3 channels as well as GIRK channels. As shown in Figures S9–S11, the model responses captured the experimental pharmacological data very well. In summary, we demonstrate the precision of projection-specific modeling among DA SN neurons in vitro. This implies that these computational models could also be used with confidence in simulated in vivo environments.

### In vivo rebound bursting in identified DA SN neurons

**Figure 8.**
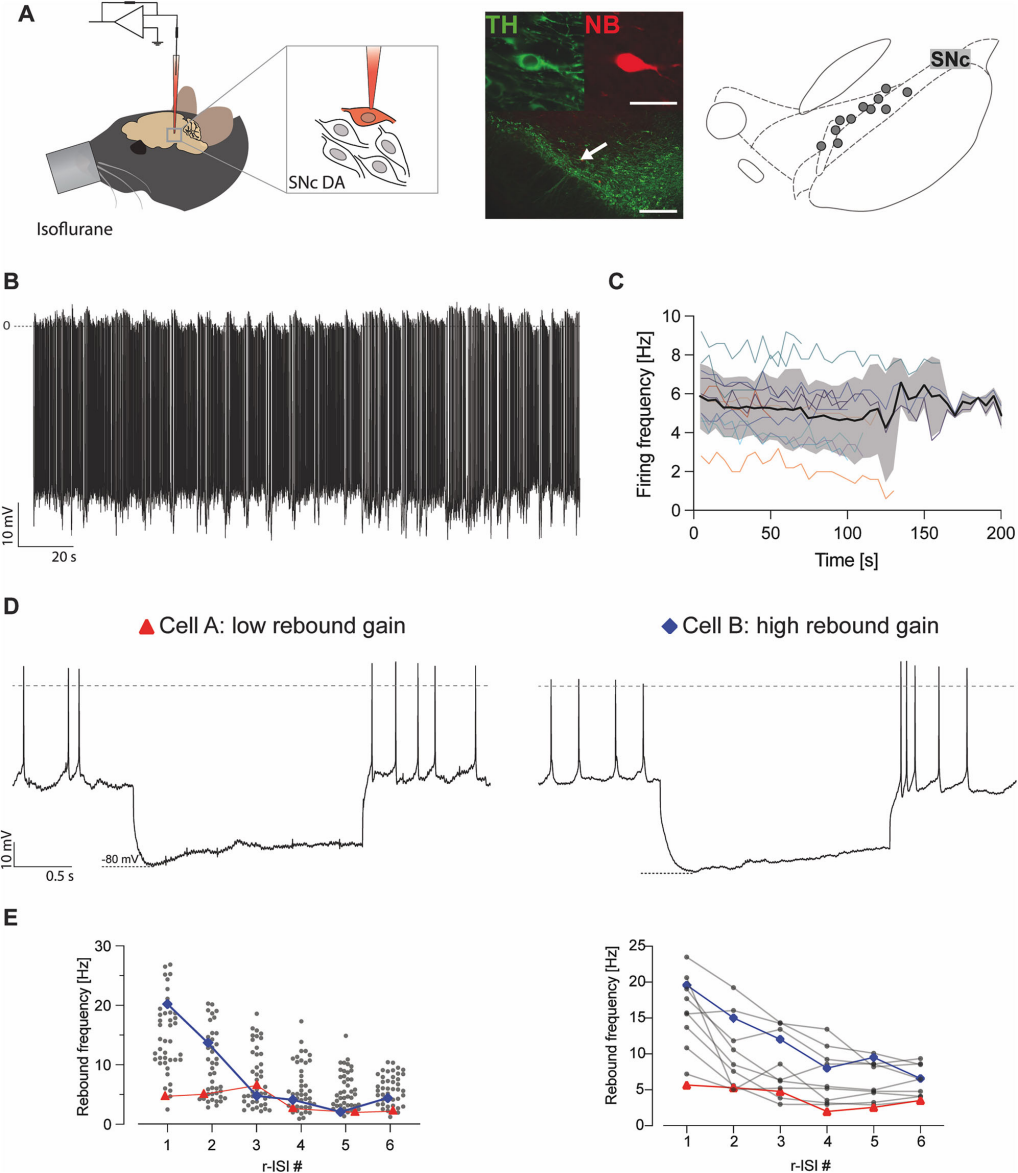
Negative current injection using in vivo whole-cell recordings reveals a wide range of intrinsic rebound excitability in DA SN neurons. ***A***, Left: Schematic illustration of the experimental approach used for in vivo patch-clamp recordings. Middle, High-magnification (60×) confocal image of immunohistochemically identified recorded neuron (top-right panel, labeled with NB, in red) and stained for tyrosine hydroxylase (top-left, TH, green). Lower-magnification (20×) confocal image locates the recorded neuron (white arrow) within the SN. Right, Anatomical mapping of all in vivo whole-cell-recorded and NB-labeled neurons (projected to the bregma −3.16 mm). Scale bars, 20 µm (top panels), 250 µm (bottom panel). ***B***, Example recording of spontaneous in vivo firing activity from a whole-cell-recorded DA SN neuron of an adult mouse under isoflurane anesthesia. The trace displays action potentials with overshoot during a stable recording for >3 min. ***C***, Time series of the mean firing rate of all recorded neurons over the entire recording length. Data from individual neurons are marked with different colors for clarity. The thick black line represents the mean value, the shaded area represents ±SD of mean. ***D***, Current-clamp recording of DA SN neurons with membrane voltage responses to 2 s injections of negative current to hyperpolarize the cells initially to −80 mV. Example traces of two DA SN neurons displaying distinct in vivo rebound firing responses to hyperpolarizing current pulses. Example traces for cell “A” displaying no increase in rebound firing (left) and cell “B” displaying significantly stronger rebound firing (right). Dashed lines indicate membrane potential at 0 mV. ***E***, Rebound frequency plotted against first six ISIs (ISI #) occurring after the end of current injection. Left, Scatterplot showing responses from all recorded traces (*n*_(traces)_ = 43; *n*_(cells)_ = 11). Right, Average rebound frequency responses for individual neurons (*n*_(cells)_ = 11; *N* = 11).

To probe whether rebound bursting is also present in a subset of DA SN neurons in the intact brain, we carried out in vivo patch-clamp recordings in anesthetized mice as previously established (Otomo et al., 2020). All cells were recorded in the SN and filled with NB for post hoc immunohistochemical and morphological identification (Fig. 8*A*). All recorded cells showed stable activity throughout the recording (Fig. 8*B*,*C*). To investigate evoked rebound excitability in vivo, we injected 2 s negative current to evoke hyperpolarization to about −80 mV. Like in vitro, DA SN neurons showed sag components in the range of ∼5–15 mV [sag (millivolt), mean: 9.4 mV, SD: 2.91; Fig. S12]. We observed a range of rebound excitability similar to our in vitro recordings, with some cells eliciting rebound bursts in the range of 15–25 Hz, while others showed no rebound frequency acceleration [rebound frequency r-ISI 1 (hertz), min, 5.68; max, 23.49; mean, 15.38; SD, 5.61; Fig. 8*D,E*]. These data demonstrate that the dynamic range of in vitro rebound excitability in DA SN neurons is maintained in the intact brain, although projection specificity could not be defined. Based on our study, we predict that in vivo rebound bursting DA SN neurons are indeed those projecting to the DLS.

### Modeling rebound properties in vivo

**Figure 9.**
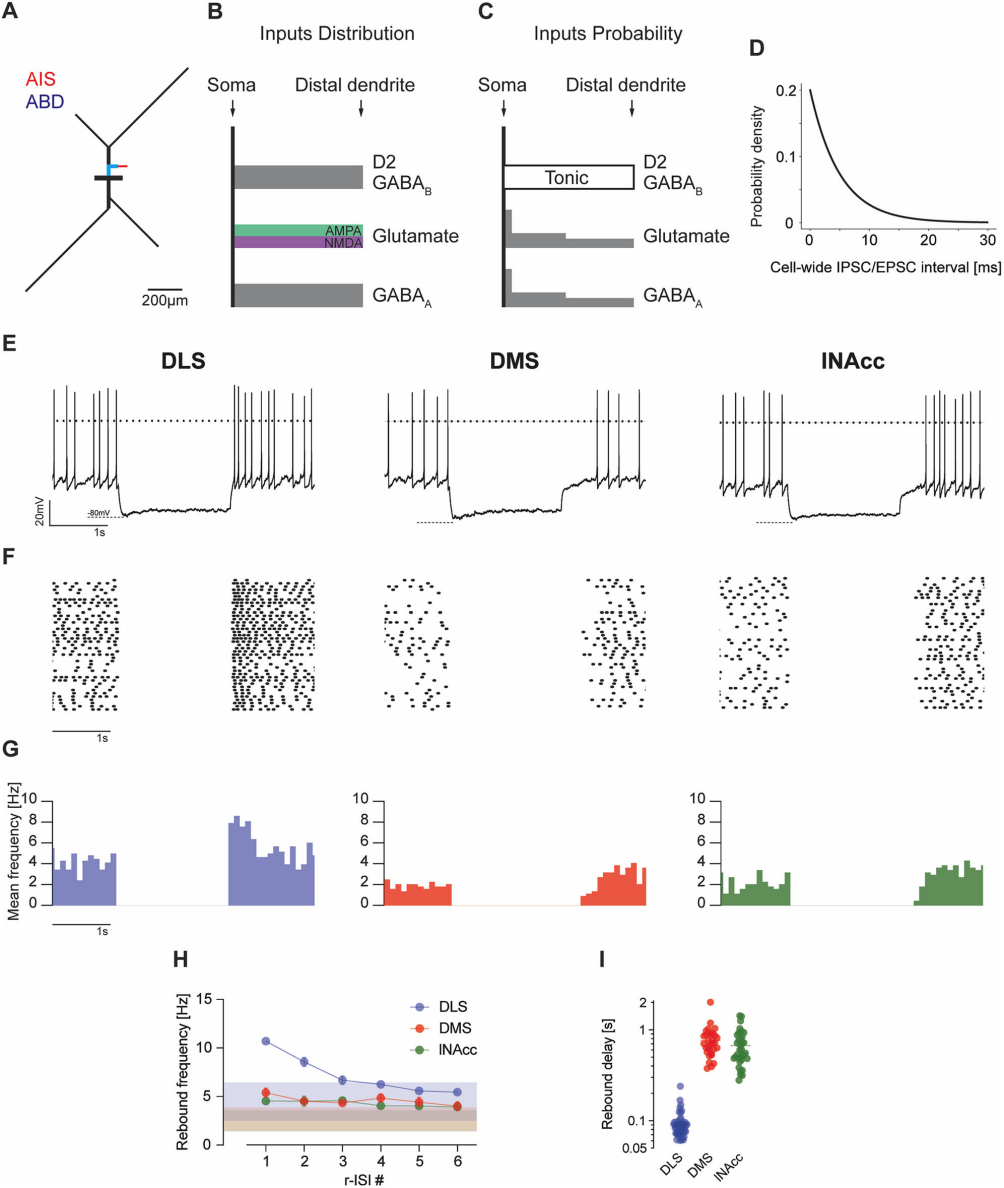
In vivo model predicts projection-specific differences are preserved under synaptic balanced state. ***A***, Schematic representation of multicompartmental model morphology with highlighted AIS (red) and ABD (blue). Compartment diameter is exaggerated relative to length for clarity. ***B***, Spatial distribution of synaptic input strength. Magnitude of E/IPSC does not vary with distance from soma. ***C***, Input probability of synaptic events. ***D***, Cell-wide GABA_A_ IPSC and glutamate EPSC probability density at each input interval (mean, 5 ms). IPSCs and EPSCs are applied independently of each other. ***E***, Representative traces for rebound from 2 s hyperpolarizing steps to −80 mV in DLS (left), DMS (mid), and lNAcc (right) projecting models. ***F***, Spike raster of 60 repetitions of the rebound protocol on the cells from (***E***). ***G***, Histogram (100 ms bin width) of raster in ***F***, excluding models with no balanced state activity. ***H***, Instantaneous frequency of the first six spikes following hyperpolarization protocol from ***E***; colored band indicates mean ± SD of instantaneous firing frequency (bin occupancy/bin width). Points/bars are mean ± SEM of *n* = 58 DLS, *n* = 46 DMS, *n* = 43 lNAcc-projecting model neurons with randomized parameters under a single hyperpolarizing protocol. ***I***, Rebound delays (log scale) of trials in ***H***.

We now used our projection-specific DA SN models to compute rebound responses of each subpopulation in a simulated in vivo environment. As shown in Figure 9*A*, this in vivo environment was characterized by the presence of a balanced and stochastic input of inhibitory (GABA_A_) and excitatory (AMPA and NMDA) synaptic inputs drawn from Poisson distributions (see Materials and Methods). In addition, we assumed a constant D2- and GABA_B_-mediated tone in vivo, represented by a constant GIRK conductance throughout the cell. These synaptic parameters were adapted (see Materials and Methods; Fig. S13) to match in vivo mean firing frequencies and variances of projection-specific DA SN neuronal activity (Farassat et al., 2019). Here, we also modeled the responses to 2 s negative current injections in silico. Figure 9*E* shows representative results for the three different projection-specific DA models. Like in our in vivo experimental results, only the DLS-projecting DA SN model displayed enhanced rebound excitability. Again, like for the in vitro simulations, we randomly chose value sets from the respective projection-specific parameter ranges to construct model neurons for each subpopulation (*n* = 60 for each projection; resulting in *n* = 58, *n* = 46, and *n* = 43 for electrically active DLS, DMS, lNAcc neurons, respectively). To probe consistency with in vivo recording results, a representative cell of each population was chosen, and the hyperpolarizing protocol was then applied in the balanced state. Figure 9*E* shows representative traces of modeling runs from these cells, while Figure 9*F* shows spike raster for all electrically active models. Figure 9*G* shows the histogram of 100 ms bins of those raster plots. As shown in Figure 9*H* only, DLS-projecting DA SN model neurons generate high rebound frequency responses above baseline firing rates. The colored band, showing the frequency mean ± SD of the respective bin sizes (normalized by bin width, number of trials to mean frequency) shows significant acceleration of the firing rate after a hyperpolarization compared with the baseline (*p* < 0.01 over 1 s following release of hyperpolarization relative to the balanced state) only in the DLS-DA neurons, while DMS and lNAcc-projecting cells display no significant rebound burst firing.

In addition, Figure 9*I* plots the rebound delays of each simulated population demonstrating that only DLS-projecting DA SN model neurons possess short rebound delays. Therefore, projection-specific in vitro models when placed in a simulated in vivo environment predict that rebound bursting in DA SN is selectively generated by the DLS-projecting subpopulation. Although our in vivo patch recordings were not projection-defined, DA neurons recorded with high rebound gains are plausible candidates for DLS-projecting neurons. In summary, our projection-specific DA SN models not only describe the electrophysiological properties of isolated neurons in vitro well, but also make useful predictions for their activity in the intact brain.

## Discussion

Our study provides evidence that DLS-DA SN neurons are unique among DA SN neurons. In addition to our previous work demonstrating that Ca_v_1.3 channels accelerate firing only in DLS-DA SN neurons (Shin et al., 2022), we here show that in the same DA subpopulation, T-type Ca^2+^ (Ca_v_3) channel selectively enabled rebound bursting in vitro and give strong evidence that this electrophysiological phenotype is also preserved in vivo. Our results are in accordance with previous studies by the Khaliq lab (Evans et al., 2017, 2020; Tarfa et al., 2017), who showed that only a subpopulation of DA SN neurons displayed strong rebound excitability sensitive to T-type Ca^2+^ (Ca_v_3) channel inhibition. Optogenetic stimulation of striosomal projection neurons selectively elicited rebound bursting activity in a calbindin-negative and Aldh1a1-positive subpopulation of DA SN neurons (Evans et al., 2020). That subpopulation clearly maps onto the DLS-projecting population identified in our study and suggests that sufficient hyperpolarization can be achieved to elicit rebound bursting in response to striosomal activity in vivo. The striosomal projections have a prominent GABA_B_ receptor-mediated component that targets dendrites (Crittenden et al., 2016) extending into the pars reticulata (SNr). The GABA_B_ component reverses at the Nerst potential for K^+^ and is therefore well suited to enable rebounding (Lacey et al., 1988). While studies from Khaliq lab did not compare different axonal projections, they reported a molecular marker profile (Aldh1a1-positive, calbindin-negative) associated with rebound bursting DA neurons. Indeed, directly comparing molecular marker expression profiles in projection-defined DA SN population demonstrated that DLS-projecting DA SN neurons are characterized by Aldh1a1+ and calbindin− profile. Here, we have not attempted to define the molecular identity of the T-type Ca^2+^ (Ca_v_3) channels in the DLS-projecting DA SN population. However, previous data including the nickel (Ni^2+^) sensitivity of native DA SN T-type channels (Wolfart and Roeper, 2002), quantitative single-cell expression data (Poetschke et al., 2015), and single-cell transcriptomics (Saunders et al., 2018) strongly suggest that native T-type channels in DLS-projecting DA SN neurons are mainly mediated by Ca_v_3.1 (α1G subunit) channels. While Ca_v_3.1 (CACNA1G) gain-of-function mutations have been associated with cerebellar ataxia and epilepsy (Weiss and Zamponi, 2020), a recent study highlighted atypical parkinsonism as an additional clinical feature of a CACNA1G channelopathy (Martínez-Villota et al., 2023).

### Ionic mechanisms of gain and timing control of rebound bursting in DLS-projecting DA SN neurons

Neuronal discharge patterns like intrinsic bursting are orchestrated by the coexpression and functional coupling of ion channels. Given the degeneracy and redundancy among ion channels, multiple combinations of ion channels are expected to enable intrinsic bursting, best analyzed in the crab somatogastric ganglion (Calabrese and Marder, 2025). Thus, we expected multiple channels to control selective rebound bursting in a DA SN subpopulation.

First, our study provided experimental evidence that Ca_v_3 channels are essential for rebound bursting in DLS-projecting DA SN neurons. Selective inhibition of Ca_v_3 channels via 70 µm NNC 55-0396 reduced the rebound frequency to pacemaker range, thereby eliminating accelerated rebound firing. In comparison, in our DLS-projecting DA SN computational model, which quantitatively replicated the twofold enhancement of rebound spiking compared with baseline pacemaker frequency, in silico inhibition of Ca_v_3 channels did not alter the maximal rebound frequency but accelerated the rebound frequency decay. As the specific DLS-projecting DA SN model was constrained by the entire baseline and pharmacological experimental dataset, the differences regarding the efficacy of Ca_v_3 inhibition might result from differences in Ca_v_3 somatodendritic density distribution and/or local calcium interactions, which are least experimentally restrained.

Second, we provide experimental evidence for a strong rebound burst gain control via SK channels. Inhibition of SK channels via 300 nm apamin boosted maximal rebound burst frequencies threefold and enhanced rebound burst duration twofold selectively in DLS-projecting DA SN neurons. Our computational model gave very similar results upon SK inhibition in silico. In the presence of apamin, additional experimental Ca_v_3 inhibition again completely removed rebound bursting, which indicates that in DLS-projecting cells, SK channels selectively sense Ca_v_3-triggered calcium signals. We previously described a similar functional coupling between Ca_v_3 and SK channels in postnatal DA SN neurons (Wolfart and Roeper, 2002). In contrast, inhibition of Ca_v_1 channels via 300 nm isradipine did not affect apamin-enhanced rebound bursting, further emphasizing the selective Ca_v_3–SK coupling. This result is also consistent with our previous work on Ca_v_1.3 channel, where SK-mediated feedback to nonrebound burst range firing was minimal in DLS-projecting DA SN neurons (Shin et al., 2022). Ca_v_3–SK coupling has been previously described in GABAergic neurons of the thalamic reticular nucleus. While this Ca_v_3–SK coupling was reduced in a mouse model of neurodevelopmental disease resulting in hypoexcitability (Ptchd1; Wells et al., 2016), in DLS-DA neurons, SK channel inhibition had the opposite effect in enhancing rebound bursting. This might predict a hyperdopaminergic phenotype in the Ptchd1 mouse model.

Third, in line with previous work (Amendola et al., 2012; Tarfa et al., 2017), we found that K_v_4.3 (A-type K^+^) channels play a key role in regulating rebound delay in DLS-DA neurons. Selective inhibition of these channels using 1 µm AmmTx3 significantly shortened the delay, highlighting their contribution to controlling the timing of postinhibitory excitability. Furthermore, Kv4.3 inhibition was the only manipulation to completely remove the projection-specific differences among the three studied DA SN subpopulations regarding not only rebound excitability but also the baseline pacemaker rate. These results emphasize the key role of differential K_v_4.3 channel properties for functional diversity of DA subpopulations.

Fourth, selective inhibition of HCN channels by 25 µm ivabradine did not affect rebound gain but prolonged the onset of rebound firing in DLS-projecting cells, not yet tested in the model. In this sense, HCN channels control timing, but not gain of rebound bursting, which might be relevant for effective postinhibitory synchronization among DLS-projecting DA SN neurons.

In addition, we also found that G_αi_-coupled GPCRs like D2 and GABA_B_ receptors dampened rebound bursting via GIRK channel activation. These experimental results were also seen in the projection-specific computational model (compare Fig. 6 and Fig. S11). Desensitization of G_αq_-coupled GPCRs such as M5R and α1-adrenergic receptors used under control conditions further stabilized the rebound bursting phenotype. In essence, DLS-projecting DA SN neurons possess a unique module of conductances that enables rebound bursting with flexible gain and timing control. To study the rebound properties in the intact brain, we carried out in vivo patch-clamp recordings as previously established (Otomo et al., 2020). As a combination with retrograde labeling was not yet feasible, we assumed that only a DA subpopulation—those that project to DLS—would display rebound bursting. Indeed, we found that DA SN neurons displayed a spectrum of rebound excitability with ∼50% showing clear rebound bursting above the in vivo pacemaker range. These results demonstrated that the intrinsic differences in rebound properties are also present in the intact brain. We again turned to modeling to probe the behavior of projection-specific DA SN models in an in silico in vivo state. When assuming a constant probabilistic barrage of balanced excitation and inhibition as well as tonic neuromodulatory tone, we observed baseline firing frequencies and variability (CV) very similar to in vivo patch-clamp recordings (and previous projection-specific juxtacellular in vivo recordings; Farassat et al., 2019). Upon injection of hyperpolarizing currents, both—experimental in vivo recordings and in vivo simulations—gave similar sag responses. Importantly, only DLS-projecting DA SN models displayed strong rebound bursting under these simulated in vivo conditions, in the similar range to the high rebound excitable subgroup of in vivo recorded DA SN neurons. Thus, our data provide support for the notion that also in vivo only those projecting to DLS possess significant rebound bursting above the pacemaker frequency range. This biophysical property provides a unique responsiveness to disinhibitory signals.

### Limitations of the study

Our study has several limitations. Regarding in vitro electrophysiology, we have not carried out a detailed comparison of Ca_v_3 currents in the three projection-specific DA SN neuron populations to validate a key assumption of the models. However, the differential responses to Ca_v_3 inhibition—both experimentally and computational—are fully consistent with our interpretation. Also, some of the in vivo model assumptions, e.g., somatodendritic density distributions of Ca_v_3 channels, are plausible but have not been explicitly validated for projection-defined DA neurons. We believe that these model's assumptions are nevertheless valid, as they were constrained by the entire experimental and pharmacological dataset. As we have not systematically explored the entire parameter space of our already complex models, we cannot fully exclude that alternative solutions might also be consistent with the experimental dataset. However, by the standards of published computational models, they have been selected under a high level of constraints.

Regarding the in vivo data, the obvious omission is the lack of projection-specific identification for in vivo patched DA SN neurons, which needs further method development. Also, it would be relevant to carry out the in vivo patch experiments in awake animals to enable probing the properties of spontaneous rebound bursts of DA SN neurons in behaviorally relevant network states.
